# 18β‐Glycyrrhetinic Acid and a Nano‐Liposomal Formulation Alleviate Depression‐Like Behaviors via the Microglial mTOR‐Autophagy‐NLRP3 Axis

**DOI:** 10.1002/advs.202523258

**Published:** 2026-03-12

**Authors:** Hua Gan, Haitao Yuan, Wenjun Zhu, Xiaokang Xie, Shen Zhou, Wenzhi Hao, Xiaowei Mo, Lian Yang, Xiaojuan Li, Junshan Liu, Lijuan Deng, Jiaxu Chen

**Affiliations:** ^1^ Guangzhou Key Laboratory of Formula‐Pattern Research Center School of Traditional Chinese Medicine and State Key Laboratory of Bioactive Molecules and Druggability Assessment Jinan University Guangzhou P. R. China; ^2^ Department of Scientific Research and Education Fangchenggang Hospital of Traditional Chinese Medicine (Fangchenggang Hospital of Guangxi University of Traditional Chinese Medicine) Fangchenggang P. R. China; ^3^ Center for Drug Research and Development Guangdong Provincial Key Laboratory for Research and Evaluation of Pharmaceutical Preparations Guangdong Pharmaceutical University Guangzhou P. R. China; ^4^ School of Traditional Chinese Medicine, Basic Research Center of Excellence for Integrated Traditional and Western Medicine for Qingzhi Diseases Southern Medical University Guangzhou P. R. China; ^5^ School of Traditional Chinese Medicine Beijing University of Chinese Medicine Beijing P. R. China

**Keywords:** 18β‐glycyrrhetinic acid, mTOR, microglia, nano‐delivery, NLRP3

## Abstract

The limited efficacy and slow onset of current antidepressants underscore the urgent need for novel therapeutic strategies. Here, we established a novel zebrafish inflammation‐based screening model and identified 18β‐glycyrrhetinic acid (18β‐GA) as a potent anti‐inflammatory candidate. In a chronic social defeat stress (CSDS) mouse model, 18β‐GA demonstrated significant antidepressant effects, which were associated with attenuated neuroinflammation and a shift in microglial polarization toward an anti‐inflammatory phenotype. Mechanistically, 18β‐GA inhibited the mTOR/p70S6K signaling pathway, leading to the restoration of autophagy and subsequent suppression of NLRP3 inflammasome activation in microglia. Using a transwell co‐culture system, we further confirmed that 18β‐GA protects neurons from microglia‐mediated inflammatory injury. To overcome pharmacokinetic limitations, we developed a nanoliposomal formulation (Nano 18β‐GA) that achieved rapid brain accumulation within 0.5 h, as visualized by time‐dependent in vivo imaging. Remarkably, a single administration of Nano 18β‐GA produced significant antidepressant effects, maintained the original mechanism of action, and exhibited a favorable biosafety profile. Together, our work delineates a translational pipeline from natural product discovery to nano‐enabled therapy, offering a rapidly acting strategy with substantial translational potential for depressive disorder.

## Graphical abstract

Using a novel zebrafish‐based inflammatory screening strategy, we screened and identified 18β‐glycyrrhetinic acid (18β‐GA) as a promising anti‐inflammatory candidate. We uncover a microglial mTOR–autophagy–NLRP3 axis that constitutes the mechanistic core of 18β‐GA–mediated neuroprotection. An engineered nanoliposomal 18β‐GA achieves rapid antidepressant efficacy after a single dose, with favorable biosafety and translational potential.

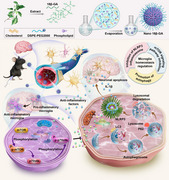

## 1 Introduction

Depression, particularly major depressive disorder (MDD), is a prevalent psychiatric disorder characterized by persistent low mood, anhedonia, and cognitive dysfunction. Affecting over 350 million individuals worldwide, depression has emerged as a leading cause of disability [1]. Its incidence is rising among adolescents, with lifetime prevalence exceeding 15% in many developed regions [2]. Despite substantial societal and economic costs, current first‐line treatments (psychotherapy and monoaminergic antidepressants such as fluoxetine, FLX) achieve remission in only ∼50% of patients, and nearly 30% develop treatment‐resistant depression [3, 4]. Moreover, clinical benefits often require 4–6 weeks and may be limited by adverse effects [5]. Collectively, these limitations highlight the urgent need for therapeutics that target alternative pathological mechanisms and provide faster, more durable efficacy.

Chronic stress‐induced neuroinflammation has emerged as a key link between psychological stress and depression [6]. Microglia, the resident immune cells of the central nervous system (CNS), dynamically polarize in response to environmental cues [6]. Under stress, microglia contribute to neural homeostasis by supporting neurogenesis, synaptic pruning, and circuit plasticity [7, 8, 9]. Chronic stress disrupts this equilibrium, however, driving microglial hyperactivation and shifting their phenotype toward a pro‐inflammatory M1‐like state [10, 11]. This transition is characterized by excessive release of inflammatory mediators, including IL‐6, TNF‐α, and IL‐1β, alongside activation of the NLRP3 inflammasome, which together amplify neuroinflammation and neuronal dysfunction [10, 11]. In contrast, an anti‐inflammation M2‐like phenotype promotes resolution of inflammation and tissue repair [12]. The delicate balance between M1‐ and M2‐like microglial phenotypes emerges as a critical determinant of neuronal survival and circuit function, suggesting that strategies to modulate microglial activation states may offer novel avenues for antidepressant development.

The interplay between autophagy and neuroinflammation has recently emerged as a promising therapeutic target [13]. Autophagy, an essential cellular clearance mechanism, regulates microglial polarization and inflammasome activity by clearing damaged organelles and aggregated proteins [14]. Impaired autophagic flux contributes to NLRP3 inflammasome overactivation and sustained neuroinflammation in stress‐related disorders [15, 16]. Modulation of autophagy‐related pathways thus represents a strategically novel approach for antidepressant development.

To identify novel anti‐inflammatory candidates, we employed a novel zebrafish‐based inflammation model for the first time to evaluate 12 bioavailable compounds derived from Xiaoyaosan (XYS), a clinically used antidepressant herbal formula [17]. Among these candidates, 18β‐glycyrrhetinic acid (18β‐GA) emerged as a promising agent that is capable of attenuating neuroinflammation and promoting a shift in microglial polarization toward an anti‐inflammatory phenotype. Mechanistically, 18β‐GA inhibited the mTOR/p70S6K signaling pathway, thereby restoring autophagy and inhibiting NLRP3 inflammasome activation in microglia, ultimately conferring neuroprotection. To improve brain delivery and accelerate therapeutic onset, we further developed a nanoliposomal formulation (Nano 18β‐GA), which rapidly accumulated in the brain within 0.5 h and exerted significant antidepressant effects after a single administration. Together, these findings implicate 18β‐GA and Nano 18β‐GA as promising candidates for depression and highlight microglia‐neuron crosstalk as a novel target for antidepressant development.

## 2 Results

### 2.1 Identification of 18β‐GA as a Potent Anti‐Inflammatory Agent by Phenotypic Screening in Zebrafish

**FIGURE 1:**
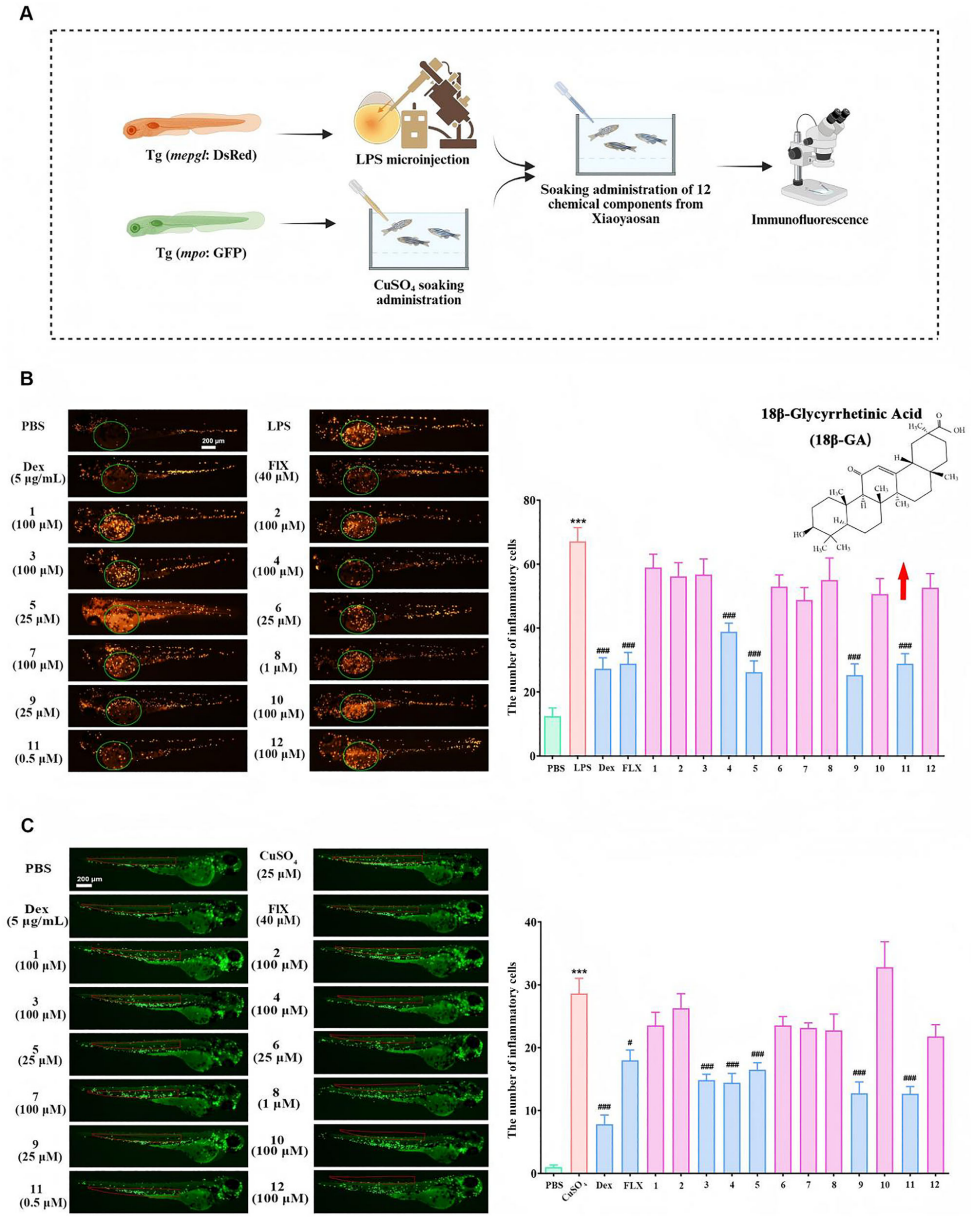
18β‐GA is a potent anti‐inflammatory agent in zebrafish. A) Schematic overview of the experimental workflow in zebrafish. B, C) Representative images and quantification of the phenotypic screen of 12 small‐molecule compounds for anti‐inflammatory activity in zebrafish subjected to LPS microinjection (B) or CuSO_4_ immersion (C). Scar bar: 200 µm. Data are presented as the mean ± SD (n ≥ 4). ^***^ *p* < 0.001 vs. PBS; ^#^ *p* < 0.05 and ^###^ *p* < 0.001 vs. model using GraphPad Prism 8.0 with one‐way ANOVA followed by Tukey's post hoc test.

To establish zebrafish models that capture the inflammatory aspects of depression, we employed two complementary paradigms: lipopolysaccharide (LPS) challenge and copper sulfate (CuSO_4_) exposure. LPS, a well‐established inflammogen, is known to induce systemic inflammation and can induce depression‐like behaviors in rodents through neuroimmune activation, including microglial stimulation and pro‐inflammatory cytokine release [18]. In parallel, CuSO_4_ serves as a chemical stressor that exacerbates oxidative stress and triggers robust neuroinflammation in zebrafish (Figure 1A) [19]. Using these models, we screened 12 blood‐absorbed bioactive compounds derived from XYS, a traditional Chinese medicine (TCM) formula widely used for its clinical antidepressant effects. In the LPS‐induced inflammation model, 18β‐GA (11) exhibited significant anti‐inflammatory efficacy at a low concentration of 0.5 µm (Figure 1B). This potent activity was reproducible in the CuSO_4_‐induced model, in which 18β‐GA again demonstrated the strongest anti‐inflammatory effect among the tested compounds (Figure 1C). Collectively, these results from two independent inflammatory paradigms indicate that 18β‐GA possesses the remarkable anti‐inflammatory activity at submicromolar concentrations.

### 2.2 18β‐GA Alleviates Depression‐Like Behaviors by Attenuating Microglia‐Driven Neuroinflammation

**FIGURE 2:**
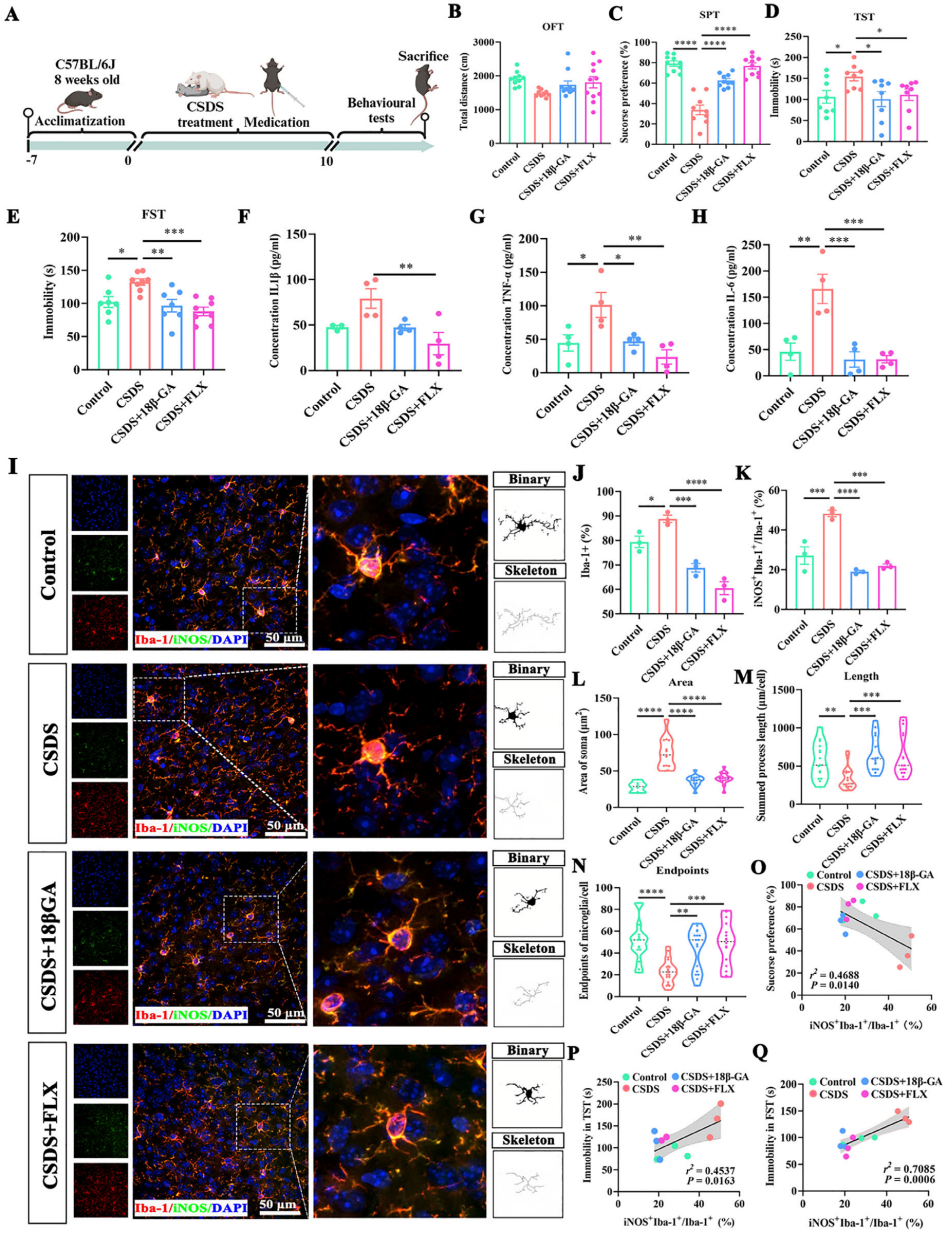
18β‐GA alleviates CSDS‐induced depressive‐like behaviors and microglial activation in mice. A) Schematic illustration of the CSDS procedure and 18β‐GA treatment. B) Total distance traveled in the OFT (n = 10). C) Sucrose preference in the SPT (n = 10). D) Immobility time in the TST (n = 10). E) Immobility time in the FST (n = 10). F‐H) Serum levels of IL‐1β (F), TNF‐α (G), and IL‐6 (H) determined by ELISA (n = 4). I) Representative immunofluorescence images of microglia labeled with Iba‐1 (red) and iNOS (green). Scale bar: 50 µm. J, K) Quantification of Iba‐1^+^ microglia (J) and the proportion of iNOS^+^ microglia (iNOS^+^Iba‐1^+^/Iba‐1^+^) (K) (n=3 of randomly selected regions from 10 mice per group). L–N) Morphometric analysis of microglia, including soma area (L), total process length (M), and number of endpoints (N) (n=3 of randomly selected regions from 10 mice per group). O‐Q) Correlation analyses between the proportion of iNOS^+^ microglia and behavioral outcomes, including SPT (O), TST immobility (P), and FST immobility (Q) (n = 3 of randomly selected regions from 10 mice per group). Data in (B‐H) and (J‐N) are presented as mean ± SEM. Statistical analysis for (B–H, J–N) using GraphPad Prism 8.0 with one‐way ANOVA followed by Tukey's post hoc test. Correlations in (O‐Q) were assessed using GraphPad Prism 8.0 with Spearman's rank correlation. ^*^ *p* < 0.05, ^**^ *p* < 0.01, ^***^ *p* < 0.001, ^****^ *p* < 0.0001.

To validate the antidepressant potential of 18β‐GA, we employed the classic chronic social defeat stress (CSDS) model [20]. As shown in Figure 2A, mice were exposed to CSDS for 10 days and subsequently treated with vehicle, 18β‐GA, or FLX, followed by behavioral and biochemical assessments. In the open field test (OFT), total distance traveled was comparable across groups (Figure 2B), indicating intact locomotor activity. Social interaction deficits induced by CSDS were significantly improved by 18β‐GA and FLX (Figure S1).

We next evaluated anhedonia using the sucrose preference test (SPT). Following CSDS exposure, a pronounced decrease in sucrose preference was observed, falling to only 33.74% in the model group. In contrast, 18β‐GA treatment significantly restored sucrose preference to 62.55%, nearly double that of the CSDS model group (Figure 2C). To further assess behavioral despair, we performed the tail suspension test (TST) and forced swim test (FST). CSDS‐exposed mice exhibited significantly prolonged immobility time in both tests. Administration of 18β‐GA significantly reduced immobility time in the TST (Figure 2D) and FST (Figure 2E), demonstrating a potent antidepressant‐like effect comparable to FLX.

Given that chronic stress is tightly linked to systemic inflammation, we quantified circulating cytokines. CSDS exposure significantly increased serum levels of IL‐1β, TNF‐α, and IL‐6 levels, all of which were effectively suppressed by 18β‐GA (Figure 2F–H), supporting its anti‐inflammatory action.

We next examined microglial activation in the medial prefrontal cortex (mPFC), a key region regulating emotion and cognition (Figure S2). CSDS exposure increased Iba‐1^+^ microglial density and the proportion of iNOS^+^/Iba‐1^+^ cells (Figure 2I–K), indicative of a pro‐inflammatory phenotype. Both 18β‐GA and FLX treatment markedly attenuated these changes. Morphological analysis further revealed CSDS‐induced microglial hypertrophy, characterized by enlarged soma size, reduced total branch length, and fewer process endpoints; 18β‐GA substantially reversed these morphological alterations (Figure 2L–N).

Finally, correlation analyses supported a link between microglial pro‐inflammatory status and behavioral outcomes. The proportion of iNOS^+^/Iba‐1^+^ microglia negatively correlated with sucrose preference (Figure 2O), and positively correlated with immobility time in the TST and FST (Figure 2P, Q). Collectively, these findings indicate that 18β‐GA mitigates CSDS‐induced depressive‐like behaviors, at least in part, by suppressing microglia‐driven neuroinflammation and associated maladaptive morphological remodeling.

### 2.3 18β‐GA Alleviates Depression‐Like Behaviors by Suppressing NLRP3 Activation and Promoting Autophagy in Microglia

**FIGURE 3:**
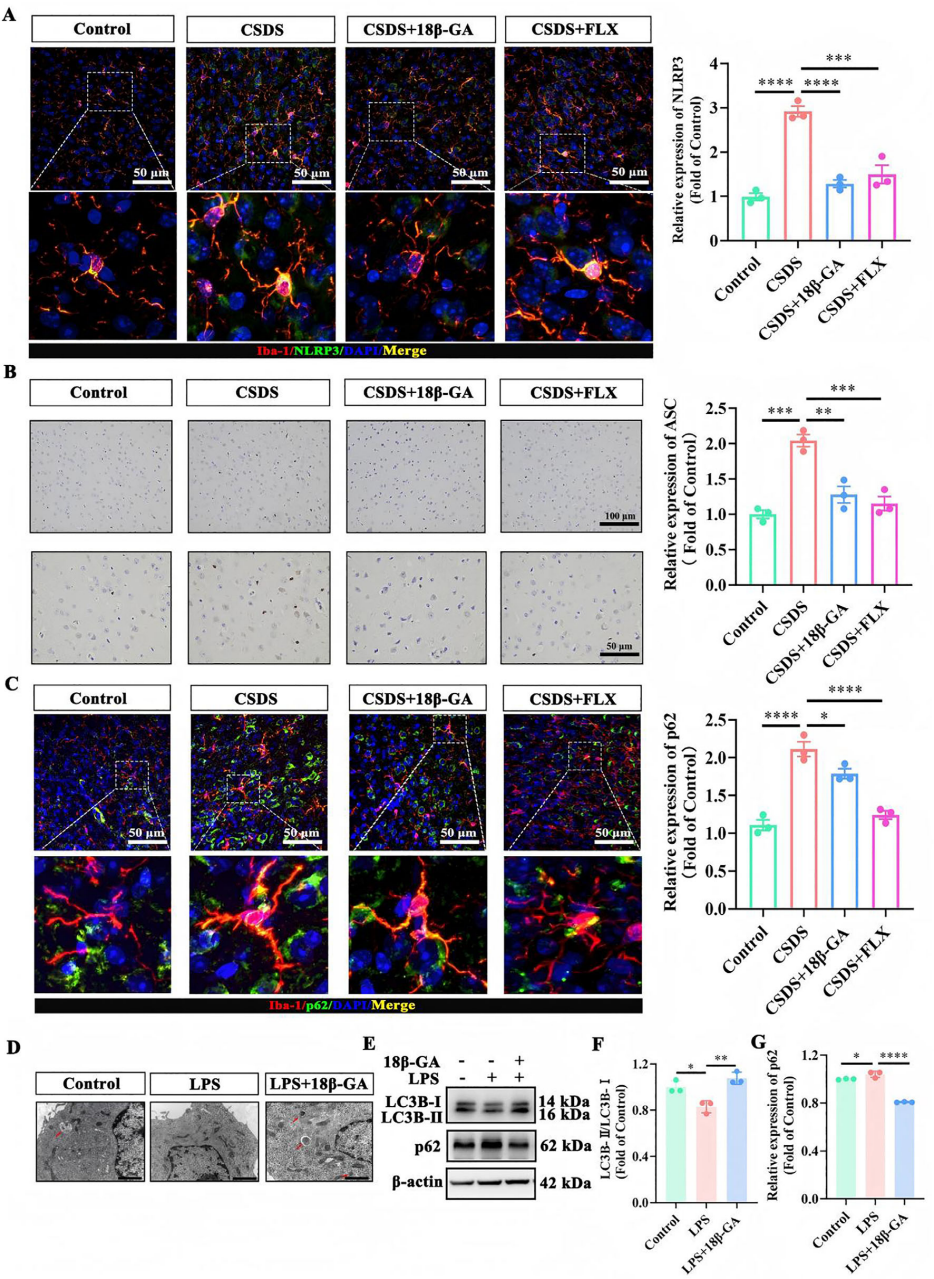
18β‐GA restores microglial autophagy and suppresses CSDS‐induced NLRP3 inflammasome activation. A) Representative immunofluorescence images and quantification of NLRP3 in the mPFC (n = 3 of randomly selected regions from 10 mice per group). Scale bar: 50 µm. B) Representative immunohistochemical images and quantification of ASC expression in the mPFC (n = 3 of randomly selected regions from 10 mice per group). Upper images: scale bar, 100 µm; lower images: scale bar, 50 µm. C) Representative immunofluorescence images and quantification of p62 in the mPFC (n = 3 of randomly selected regions from 10 mice per group). Scale bar: 50 µm. D) Representative TEM images of the microstructure in BV2 cells under inflammatory conditions, with or without 18β‐GA treatment. E) Representative immunoblots of LC3B‐I, LC3B‐II, and p62 in BV2 cells. β‐Actin served as the loading control. F, G) Quantification of LC3B‐II/LC3B‐I (F) and p62 protein levels (G) (n = 3). Data in (A–C) are presented as mean ± SEM; data in (F, G) are presented as mean ± SD. ^*^ *p* < 0.05, ^**^ *p* < 0.01, ^***^ *p* < 0.001, ^****^ *p* < 0.0001 using GraphPad Prism 8.0 with one‐way ANOVA followed by Tukey's post hoc test.

Building on the evidence that 18β‐GA suppresses microglial activation and ameliorates depressive‐like behaviors, we next investigated the underlying molecular mechanisms. The NLRP3 inflammasome is a key driver of pro‐inflammatory cytokines maturation and release [21], and its activation has been implicated in depression pathogenesis. We therefore assessed whether CSDS triggers the NLRP3 activation in the mPFC. Immunofluorescence labeling revealed a marked increase in NLRP3 fluorescence intensity in the mPFC of CSDS‐exposed mice, which was significantly attenuated by 18β‐GA treatment (Figure 3A). Since the protein ASC is essential for NLRP3 inflammasome assembly, we further performed immunohistochemical staining for ASC. Consistent with NLRP3 upregulation, CSDS robustly increased ASC levels in the mPFC, and this effect was markedly attenuated by 18β‐GA (Figure 3B), suggesting that 18β‐GA negatively regulates NLRP3 inflammasome complex formation.

We next asked whether autophagy contributes to the anti‐inflammatory mechanism of 18β‐GA. We observed pronounced expression of p62, a selective autophagy substrate that accumulates when autophagy is inhibited, in the mPFC of CSDS‐exposed mice (Figure 3C). This accumulation implies impaired autophagy, which may contribute to neuroinflammation and depressive‐like behaviors. Treatment with 18β‐GA significantly reduced p62 fluorescence intensity, indicating the restoration of autophagy.

To validate the interplay between autophagy and inflammation, we employed an LPS‐induced in vitro model in microglia. Transmission electron microscopy (TEM) revealed that under inflammatory stress, autophagic structures were scarcely detectable. However, 18β‐GA treatment restored the formation of autolysosomes, confirming its pro‐autophagic activity (Figure 3D). Western blotting analysis corroborated these findings, showing that LPS‐induced autophagy impairment, evidenced by decreased LC3B‐II/LC3B‐I ratio and elevated p62, was effectively reversed by 18β‐GA (Figure 3E–G). Additional visualization of p62 in cellular assays further supported its downregulation by 18β‐GA (Figure S3).

Taken together, these results demonstrate that 18β‐GA suppresses CSDS‐associated NLRP3 inflammasome activation and restores autophagy in microglia, providing a mechanistic basis for its potent anti‐neuroinflammatory and antidepressant effects.

### 2.4 18β‐GA Suppresses the NLRP3 Inflammasome in Microglia by Inhibiting the mTOR‐p70S6K Pathway

**FIGURE 4:**
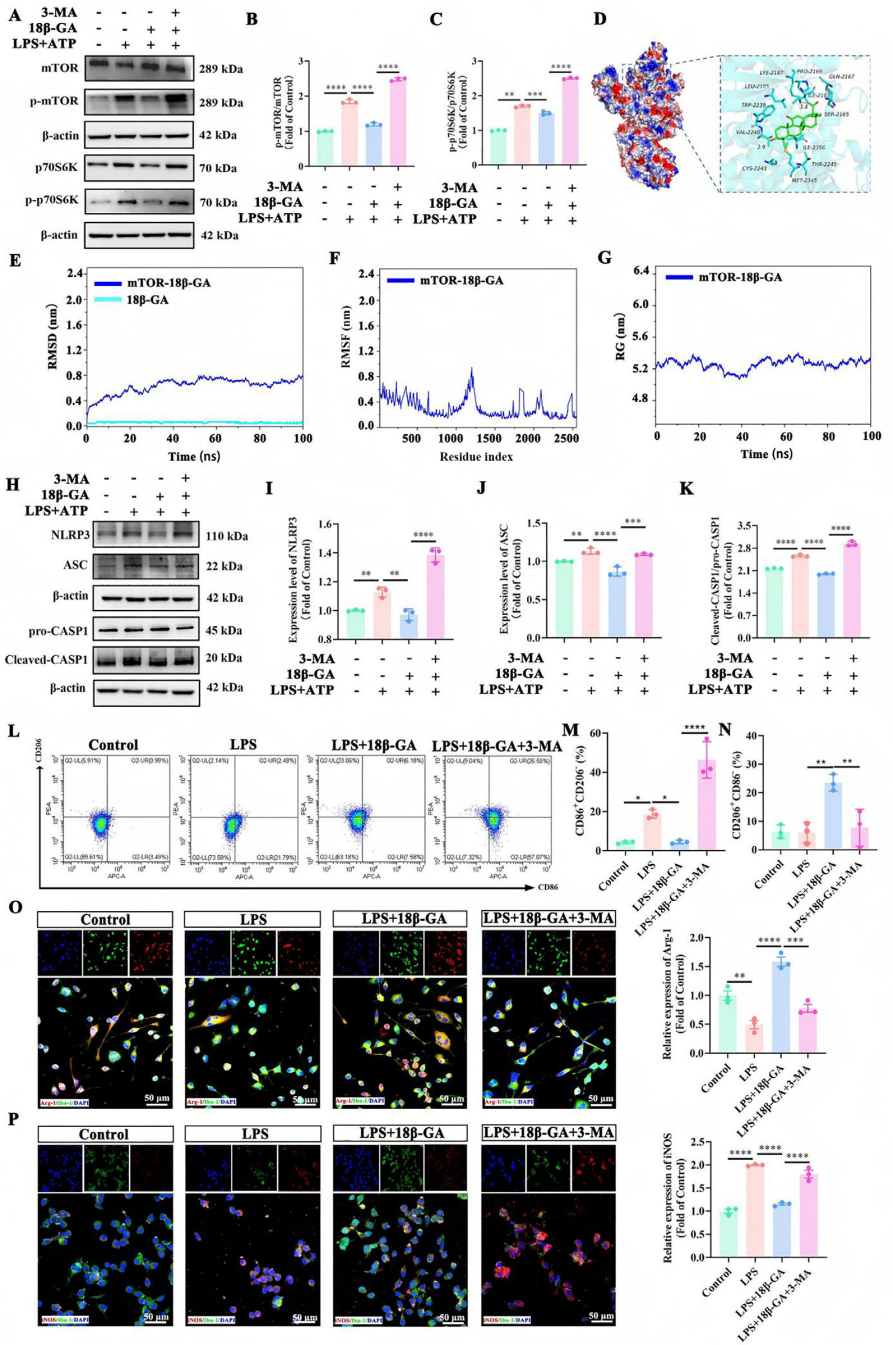
18β‐GA restores autophagy by inhibiting the mTOR/p70S6K signaling, thus suppressing NLRP3 inflammasome activation and regulating microglial polarization. A) Representative immunoblots of mTOR, p‐mTOR, p70S6K, and p‐p70S6K. β‐Actin served as the loading control. B, C) Quantification of p‐mTOR/mTOR (B) and p‐p70S6K/p70S6K (C) (n = 3). D) Predicted binding mode of 18β‐GA within the active site pocket of mTOR. E–G) Molecular dynamics simulation analyses of the 18β‐GA‐mTOR complex: RMSD (E), RMSF (F), and RG (G). H) Representative immunoblots of NLRP3, ASC, pro‐CASP1, and Cleaved‐CASP1. β‐Actin served as the loading control. I–K) Quantification of the NLRP3 (I), ASC (J), and the Cleaved‐CASP1/pro‐CASP1 (K) (n = 3). L) Representative flow cytometry plots illustrating CD86 and CD206 expression for identification of M1‐like (CD86^+^CD206^−^) and M2‐like (CD206^+^CD86^−^) microglial phenotypes. M, N) Quantification of CD86^+^CD206^−^ cells (M) and CD206^+^CD86^−^ (N) (n = 3). O, P) Immunofluorescence staining and quantification of the anti‐inflammatory marker Arg‐1 (O) and the pro‐inflammatory marker iNOS (P) (n = 3). Scale bar: 50 µm. All data are presented as mean ± SD, ^*^ *p* < 0.05, ^**^ *p* < 0.01, ^***^ *p* < 0.001, ^****^ *p* < 0.0001 using GraphPad Prism 8.0 with one‐way ANOVA followed by Tukey's post hoc test.

Having established that 18β‐GA suppresses NLRP3 inflammasome activation and restores autophagy, we next investigated upstream regulatory events. We focused on the mammalian target of rapamycin (mTOR), a canonical negative regulator of autophagy that is activated in depression models and suppresses autophagy through phosphorylation of downstream effectors such as p70S6K [22]. In LPS+ATP stimulation microglia, Western blotting showed activation of the mTOR‐p70S6K cascade, as indicated by increased p‐mTOR/mTOR and p‐p70S6K/p70S6K ratios. Notably, 18β‐GA effectively downregulated phosphorylation of both mTOR and p70S6K, decreasing their activation ratios (Figure 4A–C), demonstrating its inhibitory effect on the mTOR/p70S6K signaling axis. Consistently, pharmacological blockade of autophagy using 3‐methyladenine (3‐MA) increased phosphorylation of mTOR and p70S6K, supporting a close coupling between autophagy inhibition and mTOR pathway activation under inflammatory conditions (Figure 4A–C).

To directly characterize the interaction between 18β‐GA and mTOR, we performed molecular dynamics simulations. Molecular docking predicted a favorable binding conformation of 18β‐GA within the mTOR active‐site pocket (Figure 4D). Specifically, the C‐3 hydroxyl and C‐11 ketone groups of 18β‐GA are predicted to form hydrogen bonds with key residues in the ATP‐binding sites of mTOR, potentially inhibiting its kinase activity, whose mechanism is shared with other triterpenoids such as ursolic acid [23]. The calculated binding free energy (ΔG = −25.54 ± 1.16 kcal/mol) supported stable complex formation (Table S1). Throughout the 100 ns simulation, the complex exhibited stable, as evidenced by low Root Mean Square Deviation (RMSD) values that plateaued after ∼50 ns (Figure 4E), consistent residue Root Mean Square Fluctuation (RMSF) profiles (Figure 4F), and a decreasing Radius of Gyration (RG), suggesting enhanced structural compactness (Figure 4G). A concomitant reduction in Solvent‐Accessible Surface Area (SASA) further confirmed the stability of the complex (Figure S4). Notably, 18β‐GA maintained approximately two stable hydrogen bonds with key amino acids in the binding pocket, underpinning the specific molecular interaction.

We then assessed the functional consequences of mTOR pathway inhibition by 18β‐GA in an inflammatory cellular model. In LPS+ATP‐stimulated BV2 microglia, protein levels of NLRP3, ASC, and Cleaved Caspase‐1 (Cleaved CASP1) were markedly increased, confirming inflammasome activation (Figure 4H). 18β‐GA significantly downregulated these inflammasome components. Crucially, when autophagy was pharmacologically blocked using 3‐MA, we observed a substantial enhancement of NLRP3 inflammasome activation, reflected by elevated protein levels of NLRP3, ASC, and the Cleaved‐CASP1/pro‐CASP1 ratio compared to the LPS+ATP group (Figure 4I–K). This suggests that the anti‐inflammatory effect of 18β‐GA is dependent, at least in part, on its pro‐autophagic activity.

Given the link between inflammasome signaling and microglial inflammatory states, we further evaluated microglial polarization. Flow cytometry analysis showed that LPS promoted a pro‐inflammatory M1‐like phenotype (CD86^+^CD206^−^), which was dramatically exacerbated by 3‐MA co‐treatment (57.07%). In contrast, 18β‐GA potently suppressed M1‐like polarization (7.58%) and significantly increased the population of anti‐inflammatory M2‐like microglia (CD206^+^CD86^−^) to 23.06% (Figure 4L–N). Confocal microscopy corroborated these findings, showing that 18β‐GA decreased the pro‐inflammatory marker iNOS while increasing the anti‐inflammatory marker Arg‐1 (Figure 4O, P). Interestingly, this modulatory response of 18β‐GA was specific to the inflammatory context. In IL‐4‐induced anti‐inflammatory microglia, 18β‐GA did not suppress mTOR phosphorylation and instead upregulated NLRP3 and Cleaved‐CASP1, whereas 3‐MA activated mTOR signaling (Figure S5). Together, these data support a model in which 18β‐GA binds to and inhibits mTOR under pro‐inflammatory conditions, thereby restoring autophagy and subsequently suppressing NLRP3 inflammasome activation and M1‐like polarization in microglia. This mTOR‐autophagy‐NLRP3 axis likely underpins the anti‐neuroinflammatory and antidepressant‐relevant actions of 18β‐GA.

### 2.5 18β‐GA Alleviates Neurotoxicity In Vitro and In Vivo

**FIGURE 5:**
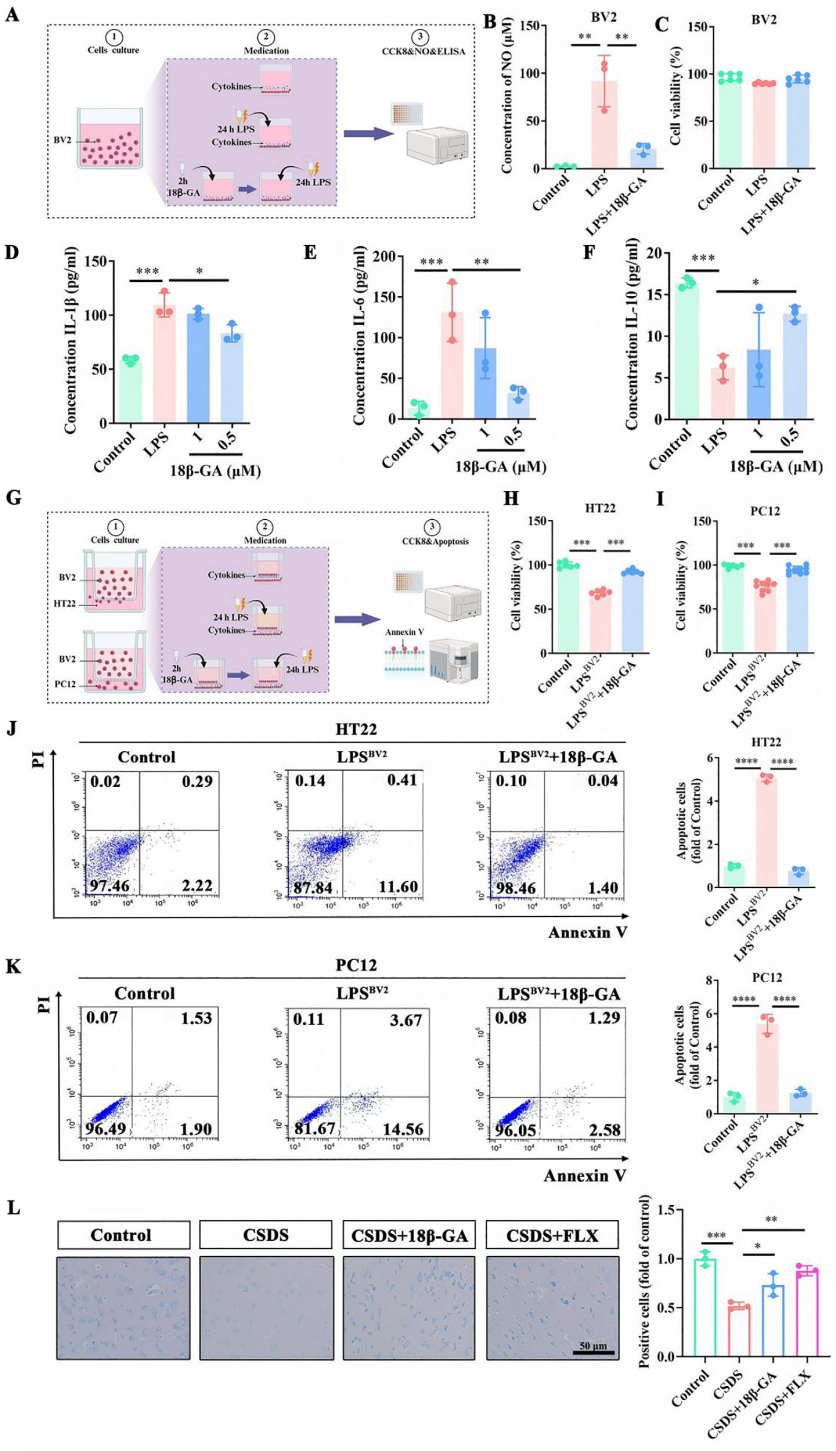
18β‐GA alleviates neurotoxicity. A) Experimental workflow of the BV2 microglia stimulation and 18β‐GA treatment. B, C) NO concentration in culture supernatant (B) and BV2 cell viability (C) following the indicated treatments (n = 3). D‐F) Cytokine levels of IL‐1β (D), IL‐6 (E), and IL‐10 (F) in culture supernatants (n = 3). G) Schematic diagram of the transwell co‐culture system between BV2 microglia and HT22 or PC12 neurons. H, I) Viability of HT22 (H) and PC12 (I) cells after co‐culture with conditioned BV2 cells (n = 3). J, K) Apoptosis rates of HT22 (J) and PC12 (K) cells assessed by flow cytometry (n = 3). L) Representative Nissl staining of the mPFC and quantification of Nissl‐positive neurons (n = 3 of randomly selected regions from 10 mice per group). Scale bar: 50 µm. Data in (B–F, H–K) are presented as mean ± SD. Data in (L) are presented as mean ± SEM. ^*^ *p* < 0.05, ^**^ *p* < 0.01, ^***^ *p* < 0.001, ^****^ *p* < 0.0001 using GraphPad Prism 8.0 with one‐way ANOVA followed by Tukey's post hoc test.

Having established that 18β‐GA suppresses neuroinflammation via the mTOR/p70S6K/NLRP3 axis, we next asked whether this anti‐inflammatory effect translates into neuroprotection, which is closely linked to antidepressant efficacy. It is well documented that inflammatory microglia contribute to neuronal damage and loss, ultimately impairing neural circuit function [24]. To address this, we polarized BV2 microglia toward a pro‐inflammatory state using LPS and evaluated their response to 18β‐GA treatment over 24 h (Figure 5A). LPS stimulation significantly increased nitric oxide (NO) release without affecting cell viability, whereas 18β‐GA markedly reduced NO levels in the culture medium (Figure 5B, C). Moreover, while LPS triggered substantial secretion of the pro‐inflammatory cytokines IL‐1β and IL‐6, 18β‐GA suppressed their release in a concentration‐dependent manner (Figure 5D, E). At 0.5 µM, 18β‐GA also significantly enhanced the production of the anti‐inflammatory cytokine IL‐10 (Figure 5F).

To determine how microglial inflammatory status affects neuronal survival, we employed a transwell co‐culture system to model microglia–neuron crosstalk (Figure 5G). LPS‐activated BV2 cells markedly reduced the viability of both mouse hippocampal HT22 neurons and rat PC12 cells, with survival rates dropping to 68.59% and 76.10%, respectively. Importantly, 18β‐GA treatment significantly restored neuronal survival, increasing viability to 92.51% in HT22 and 94.75% in PC12 cells (Figure 5H, I). Of note, low concentrations of LPS alone did not directly impair neuronal survival (Figure S6), indicating that soluble inflammatory mediators released from microglia, rather than LPS itself, were primarily responsible for disrupting the neuronal microenvironment.

We further investigated whether microglia‐driven inflammation triggers neuronal apoptosis. Flow cytometric analysis revealed that LPS‐stimulated BV2 cells strongly induced apoptosis in both HT22 and PC12 neurons. The apoptosis rate in HT22 cells reached 12.01%, nearly fivefold higher than that of the control group, but fell to 1.44% following 18β‐GA treatment (Figure 5J). A similar protective effect was observed in PC12 cells, where the apoptosis rate of 18.23% under inflammatory conditions was reduced to 3.87% with 18β‐GA (Figure 5K).

To validate these findings in vivo, we examined neuronal integrity in the mPFC using Nissl staining. CSDS‐exposed mice showed approximately a 50% reduction in Nissl‐positive neurons compared with controls. 18β‐GA robustly reversed this neuronal loss, restoring Nissl‐positive cell counts to 73.24% of the normal level (Figure 5L). Collectively, these data demonstrate that 18β‐GA confers neuroprotection by suppressing microglial pro‐inflammatory polarization and reducing the release of key cytokines (e.g., IL‐1β and IL‐6), thereby mitigating inflammation‐induced neuronal apoptosis and loss and ultimately alleviating depressive‐like behaviors.

### 2.6 Synthesis, Characterization, and In Vivo Biodistribution of 18β‐GA‐Loaded Nanoliposomes

**FIGURE 6:**
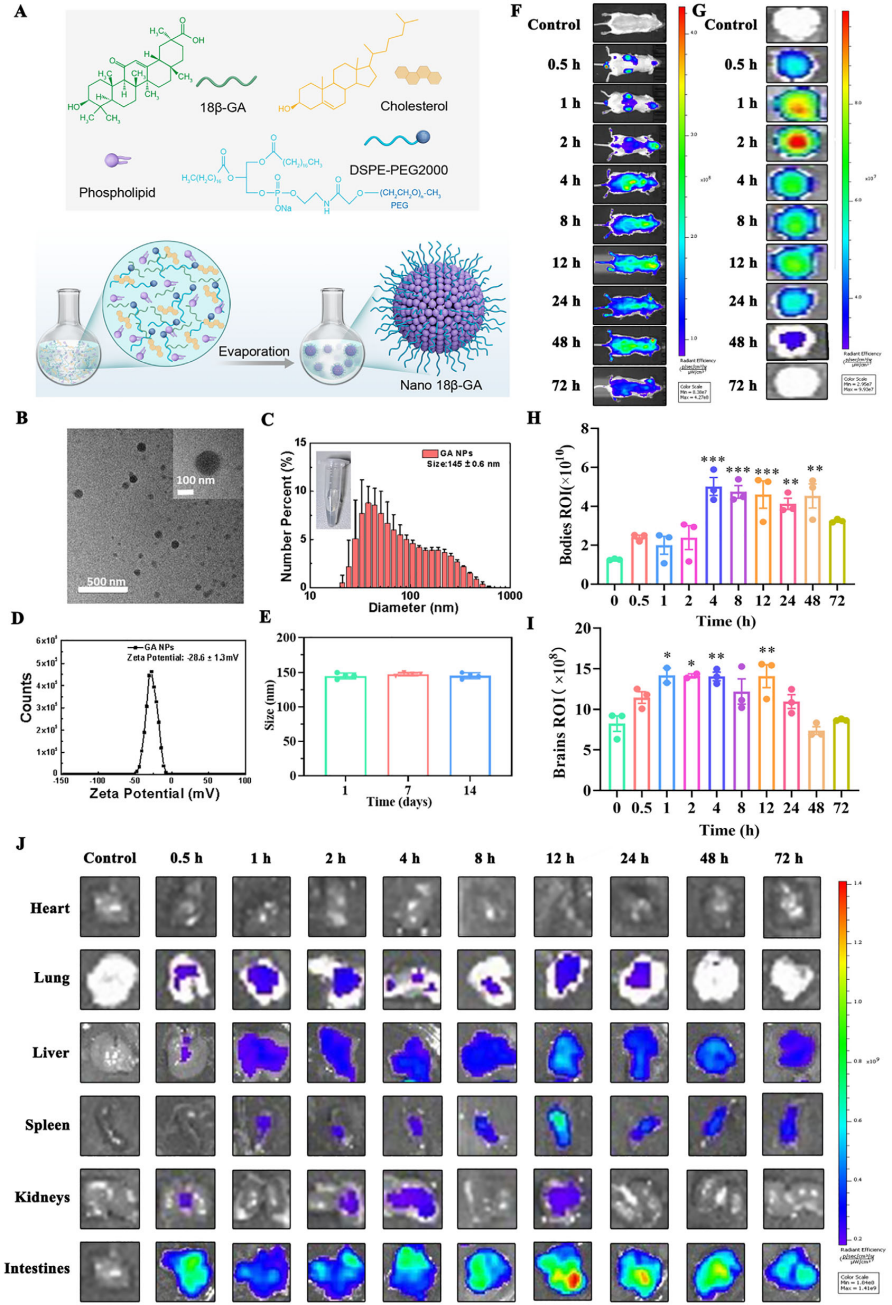
Synthesis, characterization, and biodistribution of Nano 18β‐GA. A) Schematic illustration of Nano 18β‐GA synthesis. B) TEM images of Nano 18β‐GA. The inset shows the zoomed‐in image of a representative particle. C) Hydrodynamic size distribution of Nano 18β‐GA (n = 3). D) ζ potential of Nano 18β‐GA (n = 3). E) Stability assessment of Nano 18β‐GA particle diameter measured at 1, 7, and 14 days after synthesis (n = 3). F) Whole‐body fluorescence imaging of mice at the indicated time points after intraperitoneal injection of Cy5‐labeled Nano 18β‐GA. G) Ex vivo fluorescence images of brains collected at corresponding time points. H, I) Quantification of total radiant efficiency in whole‐body (H) and brain (I) regions over time (n = 10). J) Ex vivo fluorescence imaging of major organs (heart, lung, liver, spleen, kidneys, and intestines collected at the indicated time points after injection (n = 10). Data in (C–E) are presented as mean ± SD. Data in (H, I) are presented as mean ± SEM. ^*^ *p* < 0.05, ^**^ *p* < 0.01, ^***^ *p* < 0.001 using GraphPad Prism 8.0 with one‐way ANOVA followed by Tukey's post hoc test.

To address the challenge of the slow onset of action in current antidepressant therapies, we developed a nanodelivery system to enhance the brain penetration and therapeutic timeliness of 18β‐GA. Nanoliposomes are clinically validated nanocarriers with efficient drug encapsulation, barrier‐penetrating capability, and favorable biocompatibility, exemplified by approved formulations such as Doxil (NDA 050718) and Onpattro (NDA 210922). We engineered 18β‐GA‐loaded nanoliposomes (Nano 18β‐GA) using a reverse evaporation method with phospholipids, cholesterol, and DSPE‐PEG2000 to improve aqueous dispersibility and biocompatibility (Figure 6A). Transmission electron microscopy (TEM) confirmed uniform, monodisperse nanoparticles (Figure 6B) that formed a clear colloidal solution (Figure 6C). Dynamic light scattering showed an average hydrodynamic diameter of ∼145 nm (Figure 6C), with a ζ potential of –28.6 ± 1.3 mV (Figure 6D) and excellent stability over a 14‐day period (Figure 6E).

For in vivo biodistribution, Cy5‐labeled Nano 18β‐GA was intraperitoneal injection. Whole‐body fluorescence imaging showed time‐dependent accumulation, peaking at 4 h and gradually declining by 72 h (Figure 6F, H). Notably, brain fluorescence was detected as early as 0.5 h, reaching a maximum within 2 h (Figure 6G, I), confirming rapid brain delivery. Ex vivo imaging further showed distribution in major peripheral organs, including the heart, lung, liver, kidney, spleen, and intestines, with peak signals at 12 h (except for the heart), consistent with expected pharmacokinetic profiles (Figure 6J; Figure S7).

Histological evaluation of major organs showed no obvious hemorrhage, necrosis, or tissue injury after Nano 18β‐GA treatment (Figure S8), supporting its favorable biosafety profile. These results collectively demonstrate that nanoencapsulation facilitates efficient and rapid brain delivery of 18β‐GA without apparent systemic toxicity, providing a practical strategy to accelerate antidepressant onset.

### 2.7 The Antidepressant Efficacy of Nano 18β‐GA in the CSDS Model

**FIGURE 7:**
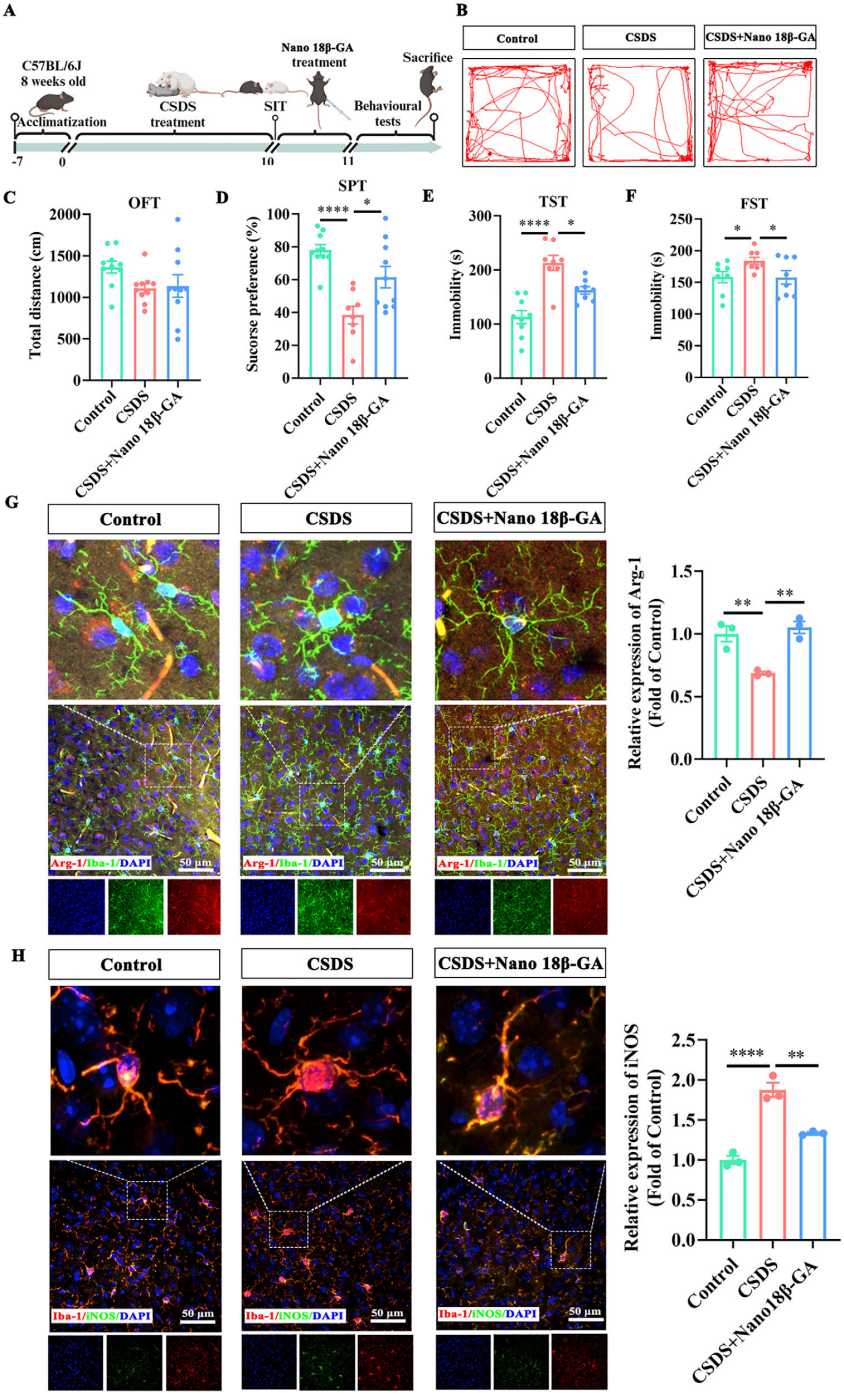
Single‐dose antidepressant efficacy of Nano 18β‐GA in the CSDS Model. A) Schematic illustration of the CSDS procedure and Nano 18β‐GA treatment timeline in vivo. B) Representative locomotion traces of mice in the OFT. C) Total distance traveled in the OFT (n = 10). D) Sucrose preference (%) in the SPT (n = 10). E, F). Immobility time in the TST (E) and FST (F) (n = 10). G, H) Immunofluorescence staining and quantification of Arg‐1 (G) and iNOS (H) in the prefrontal cortex (n = 3 of randomly selected regions from 10 mice per group). Scale bar: 50 µm. Data are presented as mean ± SEM. ^*^ *p* < 0.05, ^**^ *p* < 0.01, ^****^ *p* < 0.0001 using GraphPad Prism 8.0 with one‐way ANOVA followed by Tukey's post hoc test.

Based on the favorable biodistribution, we tested Nano 18β‐GA in the CSDS model and assessed microglial phenotypic responses. Following CSDS (Figure 7A), a single administration of Nano 18β‐GA was sufficient to reverse depressive‐like behaviors. In the OFT, Nano 18β‐GA increased exploratory behavior of the central area without altering total locomotor activity (Figure 7B, C). In the SPT, it rescued the CSDS‐induced anhedonia, increasing sucrose preference to nearly twice that of the model group (Figure 7D). Nano‐18β‐GA also significantly reduced immobility time in both the TST and FST (Figure 7E, F), demonstrating rapid antidepressant efficacy after only one administration.

This behavioral improvement was accompanied by a prompt shift in microglial inflammatory status. Immunofluorescence analysis confirmed that a single dose of Nano 18β‐GA was enough to elevate the anti‐inflammatory marker Arg‐1 (Figure 7G) and suppress the pro‐inflammatory marker iNOS (Figure 7H) in the prefrontal cortex. It also concurrently reduced NLRP3 expression (Figure S9) and decreased p62 accumulation (Figure S10). Thus, Nano 18β‐GA enables rapid‐onset, single‐dose antidepressant efficacy by promptly reprogramming the microglial autophagy–inflammasome axis.

## 3 Discussion

Natural products remain a rich source for neuropsychiatric drug discovery, yet translating promising compounds into effective CNS therapeutics is frequently limited by insufficient brain exposure and unclear cell‐type–specific mechanisms. In this study, we delineate a translational pipeline that links in vivo phenotypic discovery with mechanism‐informed validation and nano‐enabled delivery, and we develop 18β‐GA as a rapid‐acting anti‐neuroinflammatory strategy for depressive disorders. Three advances distinguish our work from prior reports. First, we introduce a novel zebrafish‐based inflammatory screening strategy that enables rapid in vivo identification of active constituents. Second, we uncover a microglial mTOR‐autophagy‐NLRP3 axis that constitutes the mechanistic core of 18β‐GA–mediated neuroprotection. Third, we engineer a nanoliposomal formulation, Nano 18β‐GA, that improves solubility and BBB penetration to produce robust antidepressant efficacy after a single administration.

Zebrafish‐based inflammation screening enables rapid in vivo identification of anti‐neuroinflammatory candidates. Using complementary zebrafish inflammatory paradigms, we identified 18β‐GA as the most active anti‐inflammatory candidate among 12 blood‐absorbed constituents derived from XYS, a TCM formula used clinically for depressive symptoms [17]. Compared with conventional mammalian‐first screening, this zebrafish strategy provides a scalable in vivo platform that integrates organism‐level pharmacology with inflammation‐relevant phenotypes, and it enables efficient prioritization of candidates with favorable bioactivity profiles. Importantly, the screening result translated beyond the aquatic model into a mammalian stress paradigm. Validation in the CSDS model demonstrated significant antidepressant efficacy, and this behavioral improvement correlated with attenuation of neuroinflammation and normalization of microglial activation state. Together, these findings support zebrafish phenotypic screening as a practical bridge between natural product libraries and mammalian disease models, and they indicate that such a strategy can accelerate the discovery of neuroimmune modulators with translational potential.

In depression, microglial polarization between pro‐inflammatory (M1‐like) and anti‐inflammatory (M2‐like) states is tightly regulated by metabolic and inflammatory signaling, in which mTOR plays a central role [25, 26]. While mTORC1 activation has been linked to M2‐like programs via STAT3 and PPARγ pathways, mTOR inhibition attenuates NF‐κB–mediated secretion of inflammatory factors such as IL‐6 and TNF‐α [27, 28]. Consistent with this, 18β‐GA promoted a shift toward an anti‐inflammatory M2‐like phenotype in microglial polarization, marked by downregulation of iNOS and CD86 and upregulation of IL‐10 and CD206. Hyperactivated mTOR signaling in the prefrontal cortex is a feature of depression [29]. contributes to suppressed autophagy and impaired clearance of damaged organelles. In line with this, 18β‐GA significantly reduced phosphorylation of mTOR and p70S6K, while increasing the LC3B‐II/LC3B‐I ratio, and decreasing p62 expression, which together indicate restoration of autophagy. Reduced p62 accumulation within Iba‐1^+^ microglia further confirmed the promotion of microglial autophagy by 18β‐GA in vivo.

Although mTOR inhibition by 18β‐GA has been described in peripheral models, such as imiquimod‐induced psoriasis and hepatic cells [30, 31], our data extend this activity to microglia in the central nervous system, which represent a key cellular node in stress‐induced neuroinflammation. Crucially, our study advances beyond mTOR modulation alone because it demonstrates an integrated link between autophagy restoration and inflammasome control. NLRP3 inflammasome activation is increasingly recognized as a hallmark of depression‐associated neuroinflammation [32]. We observed that 18β‐GA downregulated key components of the NLRP3 inflammasome, including NLRP3, ASC, and Cleaved‐CASP1, reduced NLRP3/Iba‐1 colocalization, and decreased IL‐1β secretion. Mechanistically, NLRP3 suppression may result from both direct transcriptional inhibition via mTOR signaling and enhanced degradation of inflammasome components through p62‐mediated autophagy [33]. Because NLRP3 activation can reinforce M1 polarization through NF‐κB signaling [34], its suppression likely contributes to the observed M2‐like reprogramming. Together, our results establish that microglial autophagy and NLRP3 regulation are coupled downstream of mTOR, and they identify this coupling as a critical neuroprotective mechanism in depression‐like states that has not been previously established for 18β‐GA.

Microglia–neuron crosstalk is increasingly viewed as a mechanistic interface through which immune signaling shapes synaptic and neuronal function in depression [10]. Our transwell co‐culture experiments provide functional support for this concept. Specifically, 18β‐GA preconditioning of LPS‐stimulated microglia significantly attenuates the release of pro‐inflammatory factors, rescues neuronal viability, and suppresses neuronal apoptosis. These findings suggest that the antidepressant effects of 18β‐GA are not merely associated with reduced inflammatory markers but also translate into protection of neuronal integrity under inflammatory challenge. Therefore, microglial immunometabolic reprogramming via the mTOR‐autophagy‐NLRP3 axis may represent a tractable route to preserve CNS homeostasis and mitigate depression‐related neural dysfunction. However, depression is multifactorial, and additional targets may contribute to the full therapeutic profile of 18β‐GA. Future studies that integrate genetic perturbation of pathway components, cell‐type–specific manipulations, and broader omics approaches could refine the causal hierarchy and identify complementary mechanisms.

Natural compounds frequently act through multi‐target networks. Beyond inflammation, 18β‐GA has been reported to influence metabolic transcriptional programs, such as those regulated by HNF4α, and to modulate glucocorticoid receptor signaling through dissociation of GR–HSP90 complexes, both of which are relevant to depression biology [35, 36]. While we cannot exclude contributions from additional targets, multiple lines of evidence support a dominant mTOR‐centered mechanism in microglia in our system. This conclusion is strongly supported by the molecular dynamics simulations, which confirmed a stable binding interaction between 18β‐GA and mTOR, with analyses of binding affinity and complex stability supporting direct engagement. Furthermore, pharmacological inhibition of autophagy using 3‐MA substantially reversed the suppressive effects of 18β‐GA on inflammasome activation, restoring NLRP3/ASC/cleaved CASP1 levels and counteracting the anti‐apoptotic and M2‐like–polarizing effects in microglia. These results functionally position autophagy as a required downstream effector that links mTOR modulation to inflammasome control and neuroprotection.

Nano 18β‐GA enables rapid CNS delivery and single‐dose antidepressant efficacy. A central barrier to translating many bioactive natural products into CNS therapeutics is limited solubility and inadequate BBB penetration. To address this, we engineered a nanoliposomal formulation of 18β‐GA (Nano 18β‐GA) that improves aqueous dispersibility and enhances BBB transport. Remarkably, a single administration of Nano 18β‐GA produced potent and rapid antidepressant effects in the CSDS model, accompanied by a swift rebalancing of microglial homeostasis. This rapid onset contrasts with the delayed therapeutic window typical of conventional antidepressants and highlights that nano‐enabled delivery can convert a promising natural compound into a time‐efficient intervention. Nevertheless, comprehensive evaluation of long‐term biosafety, metabolism, and pharmacokinetics, as well as optimization of dosing regimens and exploration of oral formulations, will be essential for clinical translation.

In summary, our work establishes a coherent translational route from natural product discovery to nanotherapy. We show that a zebrafish‐based in vivo screening platform identifies 18β‐GA as a top anti‐inflammatory candidate, that mechanistic interrogation reveals microglial mTOR‐dependent autophagy restoration coupled to NLRP3 suppression as a previously unrecognized neuroprotective pathway in depression, and that a rapid‐acting nanoliposomal formulation enables efficient CNS delivery and single‐dose behavioral efficacy. Together, these advances support nano‐enabled repurposing of natural compounds with limited brain availability into rapid‐acting therapeutics and provide a mechanistic framework for targeting microglial immunometabolic checkpoints in depressive disorders.

## 4 Experimental Section

### 4.1 Animals

All procedures were approved by the Laboratory Animal Welfare and Ethics Committee of Jinan University (IACUC‐20220621‐08) and conducted in accordance with the National Institutes of Health guidelines. Adult transgenic zebrafish [Tg (*mepgl*: DsRed) and Tg (*mpo*: GFP)] were maintained in a controlled aquaculture system at 28.5°C (pH 7.5; conductivity 500‐550 µS cm^−^^1^) under a 14 h light/10 h dark cycle. Fertilized embryos were obtained by natural mating and incubated at 28.5°C in egg water containing 5% methylene blue. Eight‐week‐old male C57BL/6 mice and male retired breeder CD‐1 mice were purchased from Beijing Vital River Laboratory Animal Technology Co., Ltd. Animals were housed in pairs under specific pathogen‐free conditions (20 ± 2°C; 30%–40% humidity) on a 12 h reverse light/dark cycle (lights off at 7:00 am). Food and water were provided ad libitum.

### 4.2 CSDS Procedures

CSDS was used to model chronic psychosocial stress. C57BL/6 mice were randomly assigned to CSDS or control conditions. As previously described [20], each experimental C57BL/6 mouse was co‐housed with a novel aggressive CD‐1 mouse for 10 consecutive days. The animals were separated by a perforated transparent acrylic divider that permitted continuous olfactory, visual, and auditory contact. The divider was removed for 4 min daily to allow direct confrontation. Encounters were closely monitored to ensure the occurrence of reliable failures. If defeat was not observed, the experimental C57BL/6 mouse was transferred to another aggressive CD‐1 mice and a 4‐min confrontation was repeated. Control mice were housed in pairs with CD‐1 mice for 10 days, but remained separated by the divider throughout the procedure.

### 4.3 Animal Grouping and Drug Intervention

Zebrafish drug screening. Zebrafish larvae at 3 dpf were randomly divided into 16 groups: PBS Group, LPS (5 mg/mL)/CUSO_4_ (25 µm) group, dexamethasone (Dex, 5 µg/mL) group, FLX (40 µm) Group and 12 compound groups (concentrations: 100, 100, 100, 100, 25, 25, 100, 1, 25, 100, 0.5, 100 µm). For induction of inflammation, LPS solution (1 µg/mL) was injected subcutaneously into the yolk sac. Dex, FLX, or test compounds were administered by immersion, and macrophage infiltration in swim bladders was assessed 72 h post‐injection.

Mouse intervention studies. For the mechanism intervention, 40 animals were randomly assigned to four groups (10 mice/group), with or without 10‐day CSDS. The mice in the CSDS+18β‐GA and CSDS+FLX groups received intraperitoneal injections of 18β‐GA (10 mg/kg/d) or FLX (10 mg/kg/d), respectively, for 10 days. For efficacy evaluation of Nano 18β‐GA, mice were randomized after the 10‐day CSDS procedure, and the CSDS+Nano 18β‐GA group received a single intraperitoneal injection of Nano 18β‐GA (2 mg/kg).

### 4.4 Behavioral Evaluations

Animals were acclimated to the behavioral testing chamber for at least 60 min before testing. Behavioral tests, including the SPT, OFT, TST, and FST, were performed prior to tissue collection in the same room under consistent lighting and temperature conditions. Behaviors were recorded with a video camera and analyzed using EthoVision XT (NOLDUS, Netherlands).

### 4.5 Cell culture

PC12 cells were obtained from the Cell Bank of the Chinese Academy of Sciences (CAS) and cultured in RPMI 1640 supplemented with 5% heat‐inactivated fetal bovine serum (FBS), 10% horse serum (HS), and 1% penicillin‐streptomycin (PS) at 37°C in 5% CO_2_. Nerve growth factor (NGF; 100 ng/mL, GIBCO) was added for the indicated duration. HT22 and BV2 cells (CAS) were cultured in DMEM (high‐glucose) supplemented with 10% FBS and 1% PS at 37°C in 5% CO_2_. All treatments were performed when cells reached ∼80% confluence. Cells were pretreated with 18β‐GA for 2 h, followed by LPS‐containing medium; after 24 h, all cells were collected and washed for subsequent experimental detection. All experimental cell lines were confirmed to be mycoplasma‐free by an accredited testing service (Tables S2 and S3; Figures S11–S13)

### 4.6 Immunostaining

Immunofluorescence staining of brain sections. After behavioral testing, mice were anesthetized and perfused with physiological saline followed by 4% paraformaldehyde (PFA). Brains were dissected, dehydrated, and embedded in OCT, and coronally sectioned. Images were acquired from the infralimbic (IL) region of the mPFC (AP +2.80 to +1.98 mm; ML ± 0.30 to ± 0.50 mm; DV −2.50 to −3.2 mm) [20, 37]. Sections were blocked in PBST containing 3% donkey serum and 0.3% Triton X‐100 for 1 h, incubated with primary antibodies (1:200) overnight at 4°C, washed, and incubated with secondary antibodies (1:200) for 1 h at room temperature. Nuclei were counterstained with DAPI. Images were collected using a confocal microscope (Leica) and processed with FIJI software.

Immunofluorescence staining of cells. Cells were fixed with 4% PFA for 15 min, permeabilized with PBS containing 0.5% Triton X‐100 for 20 min, and blocked with 5% bovine serum albumin for 30 min at room temperature. Cells were incubated with primary antibodies followed by fluorophore‐conjugated secondary antibodies, and images were acquired using a fluorescence microscope.

### 4.7 Western Blotting

BV2 cells (2×10^6^ cells/dish) were incubated in complete medium with or without 3‐MA (5 mM, 1 h), pretreated with 18β‐GA (0.5 µm, 2 h), and then stimulated with LPS (1 µg/mL, 24 h). Cells were lysed according to the manufacturer's instructions, and the protein concentration was determined using a BCA protein assay kit (Beyotime). Equal amounts of protein (30 µg) were separated by 10% SDS‐PAGE and transferred onto PVDF membranes. Membranes were blocked with QuickBlock blocking buffer (Beyotime) for 1 h and incubated with primary antibodies against LC3B‐I, LC3B‐II, p62, mTOR, p‐mTOR, p70S6K, p‐p70S6K, NLRP3, ASC, CASP1, and β‐actin (1:1000) overnight at 4°C (Table S4). After washing with TBST (3 × 10 min), membranes were incubated with HRP‐conjugated secondary antibodies (1:5000) for 1 h at room temperature and developed using enhanced chemiluminescence reagents (Millipore). ImageJ and GraphPad Prism v8.0 software were used to measure gray values and quantify band data.

### 4.8 Preparation and Characterization of Nano 18β‐GA and Cy5‐labeled Nano 18β‐GA

18β‐GA was dissolved in THF solution (5 mg/mL), followed by the addition of DPPC, DSPE‐PEG‐2000, and cholesterol at a weight ratio of 3:1:1. The mixture was sonicated for 30 min, added dropwise into 10 mL deionized water, and stirred for 1 h. THF was removed using a rotary evaporator, and the dispersion was stored at 4°C. For Cy5‐labeled Nano 18β‐GA, DSPE‐PEG2000‐Cy5 (50 µL; 1 mg/mL in THF) was added to the THF mixture prior to sonication, followed by the same nanoparticle preparation steps.

### 4.9 Cell Viability Assay

The protective ability of 18β‐GA against neuronal cell death induced by conditioned media from LPS‐treated BV2 cells was determined by CCK8 (APExBIO Biotechnology Co., Ltd., USA). Briefly, PC12 and HT22 cells (5 × 10^4^ cells/mL) were seeded into 96‐well plates. After co‐incubation with LPS‐treated BV2 cell supernatant, the medium was replaced with fresh medium containing 10% CCK8 and incubated at 37°C for 3 h. The absorbance was measured at 480 nm using a microplate reader (Epoch2; BioTek Instruments, Inc.).

### 4.10 Flow Cytometry Phenotype Analysis

Cells were resuspended in cell staining buffer and incubated with fluorophore‐conjugated antibodies (anti‐CD86‐APC and anti‐CD206‐PE, BioLegend) at optimized concentrations for 15–20 min on ice in the dark. The cell was washed twice with staining buffer and analyzed on a flow cytometer (Beckman Coulter, Inc.). Data were analyzed using FlowJo version 10.8.

### 4.11 Neuronal Apoptosis Detection

HT22 and PC12 cells were seeded in transwell 6‐well plates (2 × 10^5^ cells/well) and cultured overnight. After 18β‐GA pretreatment (2 h), LPS‐containing medium was added. After 24 h, cells were collected, washed, and apoptosis was assessed using a flow cytometer (Beckman Coulter, Inc.) according to the manufacturer's instructions.

### 4.12 ELISA for Cytokine Quantification

Serum levels of IL‐1β, TNF‐α, IL‐6, and IL‐10 were measured using an ELISA kit (Beyotime). Standard and samples (100 µL) were added and incubated at 37°C for 90 min. After washing, biotin‐labeled antibodywas added and incubated at 37°C for 60 min. Plates were washed, incubated with SABC solution for 30 min at 37°C, and then incubated with TMB substrate in the dark at 37°C for 15 min. The reaction was stopped, and absorbance was measured at 450 nm (Epoch2, BioTek Instruments, Inc.).

### 4.13 Transmission Electron Microscopy (TEM)

BV2 cells were collected, washed with PBS, and fixed with 2.5% glutaraldehyde overnight at 4°C. Samples were embedded, sectioned (60‐80 nm), mounted, and examined by TEM (HT7800/HT7700, Hitachi).

### 4.14 Molecular Dynamics Simulation

The protein used AMBER99SB‐ILDN force field parameters, and the small molecule ligand constructs a small molecule topology using the Sobtop program, followed by charge fitting using RESP. Select the TIP3P dominant water model, where the minimum distance between atoms in the protein and the edge of the water box is 1.0 nm, and use sodium or chloride ions to neutralize the system charge. The binding free energy of ligands and proteins was calculated using the gmxMMPBSA method of the Gromacs 2020 program.

### 4.15 Nissl Staining

Coronal paraffin sections (6 µm) of the prefrontal cortex were stained with Nissl staining solution (Servicebio) for 5 min at 37°C. Sections were rinsed in 95% ethanol for 5 min and air‐dried, then cleared twice in xylene (5 min each). After sealing with neutral balsam, the slides were observed under a light microscope (ECLIPSE E100, Nikon), and photographs of the sections were obtained.

### 4.16 In Vivo Expression of Inflammasome Markers

For immunohistochemistry, paraffin sections of the prefrontal cortex were incubated with anti‐ASC antibody for 24 h at 4°C. Stained sections were examined by a pathologist blinded to treatment conditions. Images were acquired using a microscope (ECLIPSE E100, Nikon).

### 4.17 Characterization and Biodistribution of Nano 18β‐GA

High‐resolution TEM images were acquired using a JEM‐2010HR microscope at 200 kV. Size distribution and ζ potential were obtained by a Nano ZS90 (Malvern, UK). 0.2 mL of Cy5‐labeled Nano 18β‐GA was intraperitoneally injected into mice, and the whole body and brain were recorded at the indicated time points through an in vivo fluorescence imaging system (IVIS Lumina, Series III, PerkinElmer). Heart, lung, liver, spleen, kidneys, and intestines were collected at designated time points for ex vivo fluorescence imaging.

### 4.18 Statistical Analysis

All in vitro experiments were performed at least three independent times. In vitro data are presented as the means ± standard deviation (SD), and in vivo data are presented as the means ± standard error of mean (SEM). Statistical comparisons among multiple groups were determined using GraphPad Prism v8.0 with one‐way ANOVA followed by Tukey's post hoc test. Correlations were assessed using Spearman's rank correlation. In vitro or particle characterization, n represents biological replicates. In vivo, n represents the number of animals. Exact n values and statistical tests are indicated in figure legends. The value of *p* < 0.05 was considered statistically significant.

## Author Contributions

H.G., H.Y., and W.Z. contributed equally to this work. J. C., L.D., and J.L. conceptualized the study and supervised the project. H.G., H.Y., and L.D. designed the study and prepared the initial draft and revisions of the manuscript. H.G., W.Z., and X.X. jointly performed the majority of the experiments. H.Y. assisted with the preparation and characterization of the Nano 18β‐GA at the Shenzhen laboratory. X.X. S.Z. and X. M. assisted with establishing and evaluating the animal models at the Formula‐Pattern research center. W.H. X.L and L. Y. assisted with the preliminary in vitro and in vivo experiments. J. L. contributed to zebrafish model construction and active‐ingredient screening. H.G. contributed to figure preparation and data visualization under the guidance of L.D. and J. C, who also reviewed and edited the manuscript.

## Conflicts of Interest

The authors declare no conflicts of interest.

## Supporting information




**Supporting File**: advs74756‐sup‐0001‐SuppMat.docx.

## Data Availability

The data that support the findings of this study are available in the Supporting Information material of this article.
