# Supplementary material for: 18β‐Glycyrrhetinic Acid and a Nano‐Liposomal Formulation Alleviate Depression‐Like Behaviors via the Microglial mTOR‐Autophagy‐NLRP3 Axis
Source: Adv Sci (Weinh). 2026 Mar 12;13(28):e23258. doi: 10.1002/advs.202523258 (PMC13185849; doi:10.1002/advs.202523258)
Supplement: Supplementary file 1 — Supporting File: advs74756‐sup‐0001‐SuppMat.docx. [file ADVS-13-e23258-s001.docx]

Supporting Information

18β-Glycyrrhetinic Acid and a Nano-Liposomal Formulation Alleviate Depression-Like Behaviors via the Microglial mTOR-Autophagy-NLRP3 Axis

Hua Gan^#^, Haitao Yuan^#^, Wenjun Zhu^#^, Xiaokang Xie, Shen Zhou, Wenzhi Hao, Xiaowei Mo, Lian Yang, Xiaojuan Li, Junshan Liu^*^, Lijuan Deng^*^, Jiaxu Chen^*^

Experimental section

**Materials.** Antibodies including anti-ionized calcium-binding adapter molecule 1 (Iba-1) (cat: ab283319), anti-pro-CASP1 (cat: ab207802), anti-Cleaved-CASP1 (goat anti-mouse IgG H&L (Alexa Fluor 488, cat: ab150113), and goat anti-mouse IgG H&L (Alexa Fluor 594, cat: ab150080) were purchased from Abcam. Anti-NLRP3 (cat: abs151715) and anti-ASC (cat: abs155599) were purchased from Absin. Anti-LC3B (cat: 2775), anti-SQSTM1/p62 (cat: 23214T), anti-mTOR (cat: 2972T), anti-p-mTOR (cat: 5536T), anti-p70s6k (cat: 34475T), anti-p-p70S6K (cat: 9234T), anti-Aranase-1 (Arg-1) (cat: 93668T), anti-inductible Nitric Oxide Synthase (iNOS) (cat: 13120T) and anti-β-actin (cat: 3700T) obtained from Cell Signaling Technology (CST). All secondary antibodies used for western blotting were purchased from CST. 18β-GA was purchased from Chengdu Chroma-Biotechnology Co., Ltd. LPS was purchased from Shanghai yuanye Bio-Technology Co., Ltd. 3-MA was purchased from KKL medicine. Lecithin was purchased from Aladdin. 1,2-distearoyl-sn-glycero-3-phosphoethanolamine-N-[maleimide (polyethylene glycol) 2000] (DSPE-PEG2000), Cy5-DSPE-PEG2000 and cholesterol were from Xi’an Ruixi Biological Technology Co.,Ltd. All chemical agents were used directly without further purification.

**Social interaction test (SIT).** Experimental C57BL/6 mice were placed in an open area [42 cm (W) × 42 cm (D) × 42 cm (H)] (d) × 42 cm (h)] free movement for 150 s, which contained Cubic columns of perforated acrylic, positioned directly above the center of the arena (without targets). They were then reintroduced to the arena for 150 s of free play and housed a strangely aggressive CD-1 mouse (target) in the pen. Set up a 14 cm × 24 cm rectangular area as the interaction zone (IZ) with 8cm projections around it. Mouse activity was video-monitored using Ethovision XT 14.0 software (NOLDUS EthoVision XT, Netherlands). Sus and Res mice were differentiated by the social interaction ratio (SIR), defined as (interaction time, target)/(interaction time, no target).

**Sucrose preference test (SPT).** Before the experiment, all mice were acclimatized with 1% sucrose solution for 24 h, and then each cage was placed in 1% sucrose solution and 1 bottle of normal water to drink freely for 12 h, and then the two bottles were exchanged to avoid the influence of position. Over the next 24 h, remove water and food from the mouse’s cage. During the test, each mouse was reared in a single cage, put into a bottle of 1% sucrose solution and ordinary water respectively, and the mice were allowed to drink water freely for 1 hour. The bottles were weighed before and after each test, and the sucrose preference rate was calculated according to the following formula (%): sucrose preference (%)= consumption of sucrose solution (g)/[consumption of sucrose solution (g)+ consumption of regular water (g)]× 100%.

**Open field test (OFT).** OFT was used to assess the mental excitability and state of fear and exploration of novel environments in mice. During the test, mice were placed in the central area of an opaque box (50×50×40 cm), a central area (side length: 35 cm) and an edge area were set, and they were allowed to explore freely for 5 min. Wipe the bottom of the box with alcohol before each new test to remove residual odor disturbance from the mice. All activities are monitored by cameras. Automatically calculate the frequency of entering the central area and the total distance of open field movement.

**Tail suspension test (TST).** Mice were individually suspended from the ceiling of the TST box (50×50×50 m) by distal portion of their tails with adhesive tape at approximately 1 m below the tip of the tails for 6 in. A camera was placed the front of the box and recorded the behavior of mice. The immobility time of tail-suspended mice during the last 4 in were analyzed by the software.

**Forced swimming test (FST).** The rats were placed into plastic cylinder (25 cm deep, 13 cm in diameter) filled with water at 23**-**5 ℃ up to a height of 15 cm from the base, and forced to swim for 6 min. The time of immobility behaviors (rat floating on the surface of the water, immobility of limbs, or slight body wiggling to maintain its balance) was calculated in the last 4 min.

**Histopathological examination of all organs.** The main organs of mice were fixed and then embedded in paraffin. Sections were cut to 6 µm thickness and mounted on glass slides. For histological examination, sections were stained with H&E (Servicebio) for 3 min at room temperature, and structural changes in the tissue were observed under a microscope (ECLIPSE E100, Nikon), which used to evaluate the biosafety of Nano 18β-GA.

**Statistical analysis.** All in vitro experiments were performed at least three independent times. In vitro data are presented as the means ± standard deviation (SD), and in vivo data are presented as the means ± standard error of mean (SEM). Statistical comparisons among multiple groups were determined by one-way ANOVA followed by Tukey’s post hoc test (GraphPad Prism v8.0). Correlations were assessed using Spearman’s rank correlation. In vitro or particle characterization, n represents biological replicates. In vivo, n represents the number of animals. Exact n values and statistical tests are indicated in figure legends. The value of *P* < 0.05 was considered statistically significant.

**Figures and Figure Legends**


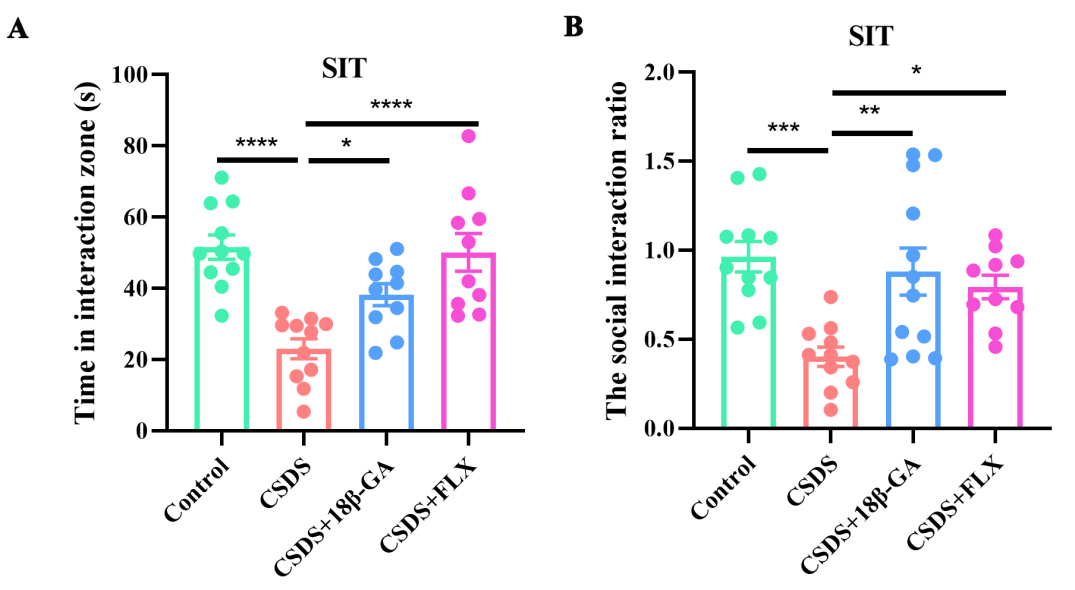


**Figure S1.** SIT of mice. A) Time in interraction zone of mice (n = 10). B) The social interaction ratio of mice (n ≥ 10). All data are presented as mean ± SEM, ^*^*P* < 0.05, ^**^*P* < 0.01, ^***^*P* < 0.001, ^****^*P* < 0.0001 using GraphPad Prism 8.0 with one-way ANOVA followed by Tukey’s post hoc test.


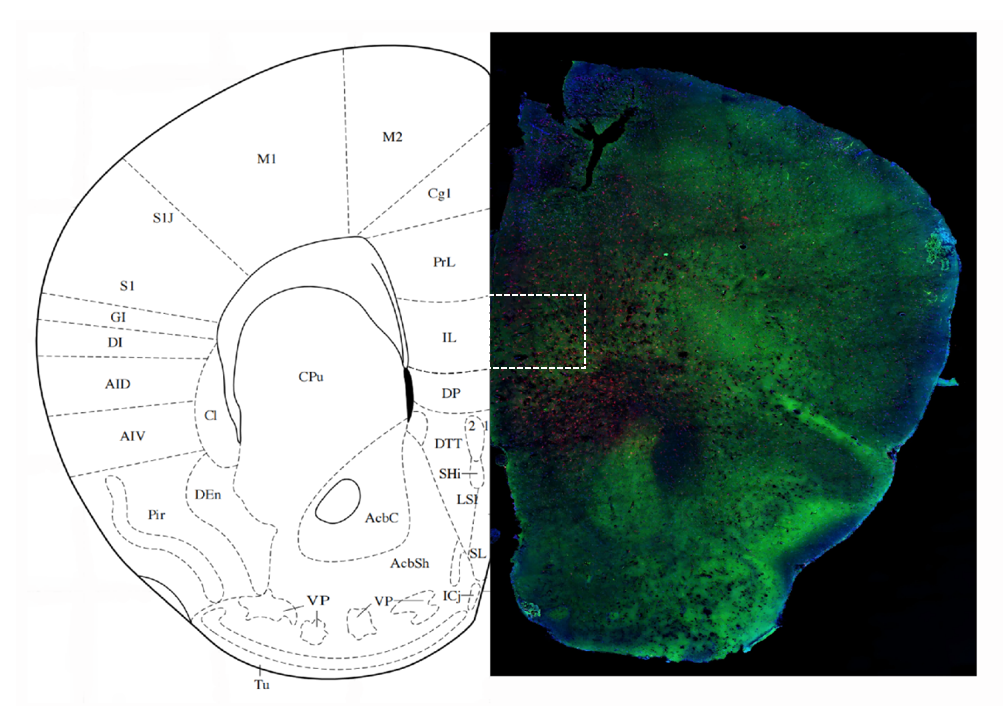


**Figure S2.** Localization of brain regions in mice.


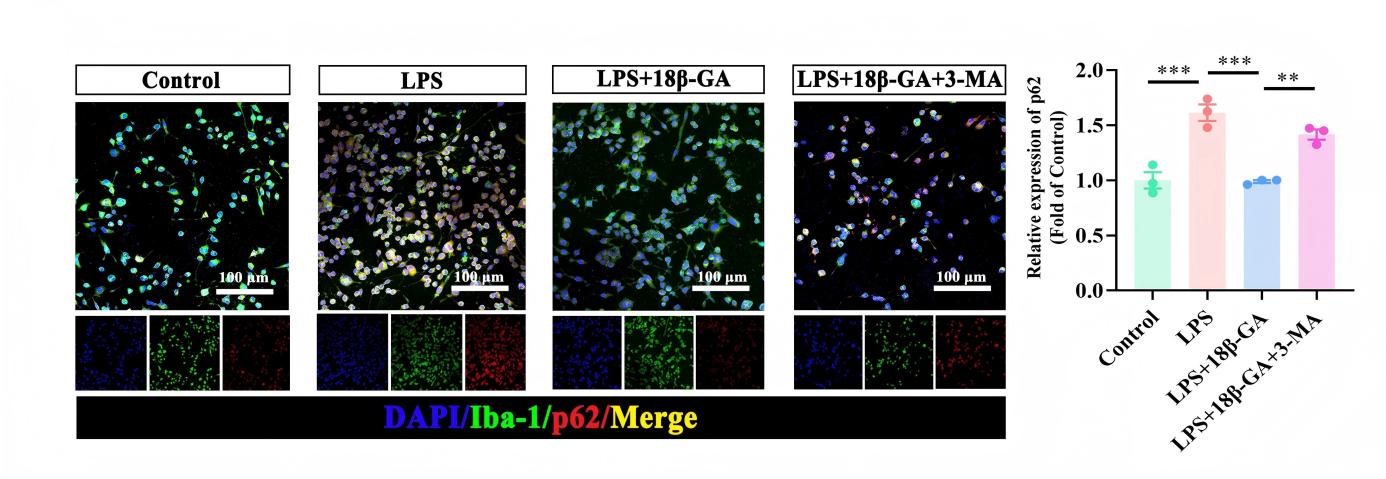


**Figure S3.** Representative immunofluorescence images and quantification of p62 in BV2 cells (n = 3). Scale bar: 50 μm. Data are presented as mean ± SD, ^**^*P* < 0.01, ^***^*P* < 0.001 using GraphPad Prism 8.0 with one-way ANOVA followed by Tukey’s post hoc test.


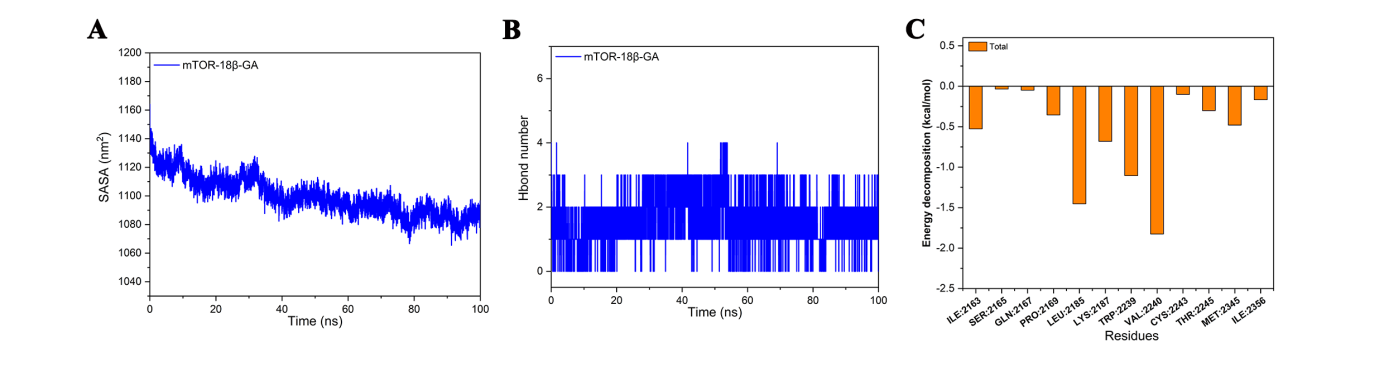


**Figure S4.** 18β-GA interacts with mTOR. A) The SASA of 18β-GA with mTOR. B) The hydrogen bond number of 18β-GA with mTOR. C) The energy decomposition of binding free energy for mTOR-18β-GA.


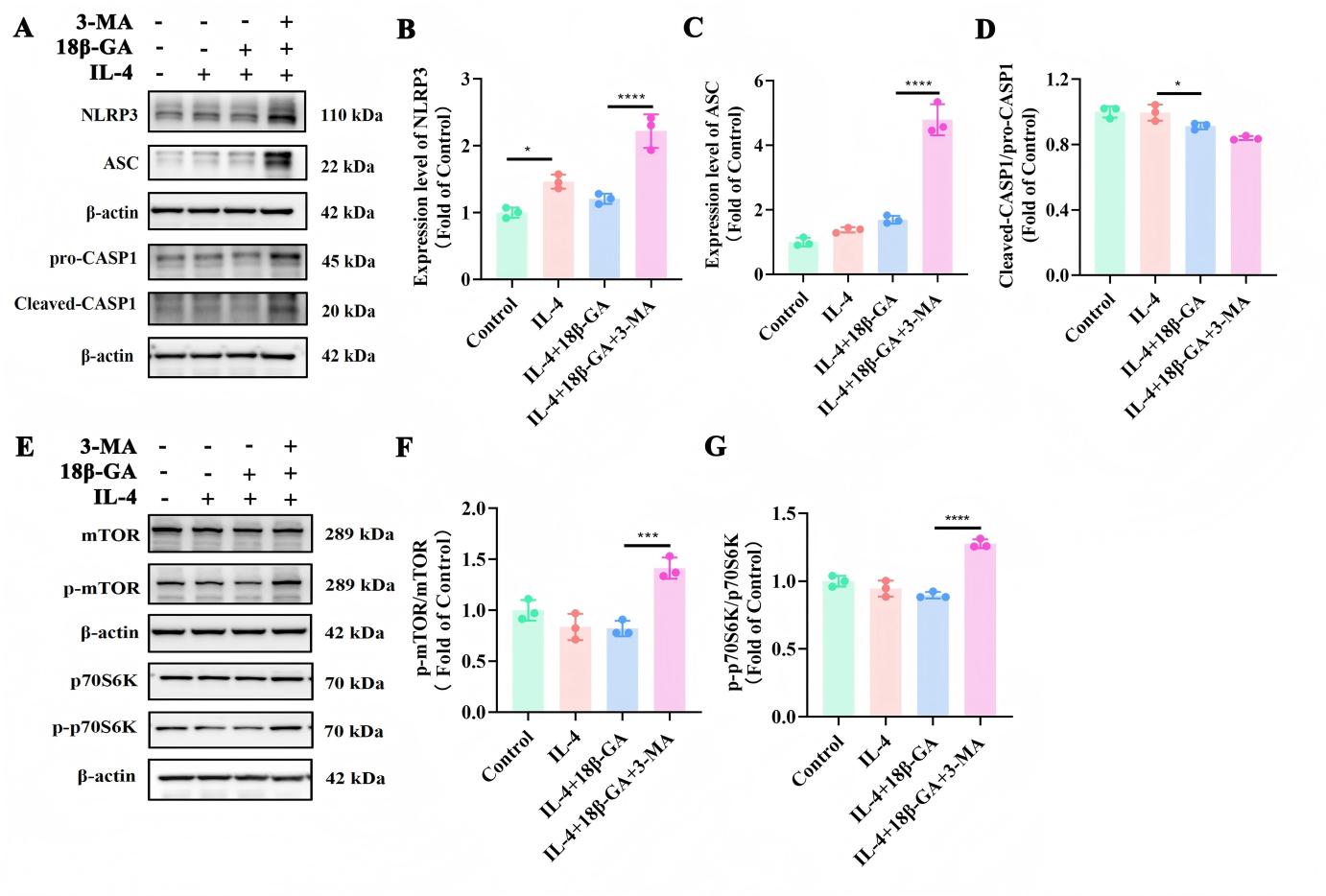


**Figure S5.** Influence of 18β-GA on mTOR Signaling and NLRP3 Inflammasome in IL-4-Induced BV2 Cells. A) Representative immunoblots of NLRP3, ASC, pro-CASP1 and Cleaved-CASP1. β-Actin served as the loading control. B**–**D) Quantification of the NLRP3 (B), ASC (C), and the Cleaved-CASP1/pro-CASP1 (D) (n = 3). E) Representative immunoblots of mTOR, p-mTOR, p70S6K, and p-p70S6K. β-Actin served as the loading control. F,G) Quantification of p-mTOR/mTOR (F) and p-p70S6K/p70S6K (G) (n = 3). All data are presented as mean ± SD, ^*^*P* < 0.05, ^***^*P* < 0.001, ^****^*P* < 0.0001 using GraphPad Prism 8.0 with one-way ANOVA followed by Tukey’s post hoc test.


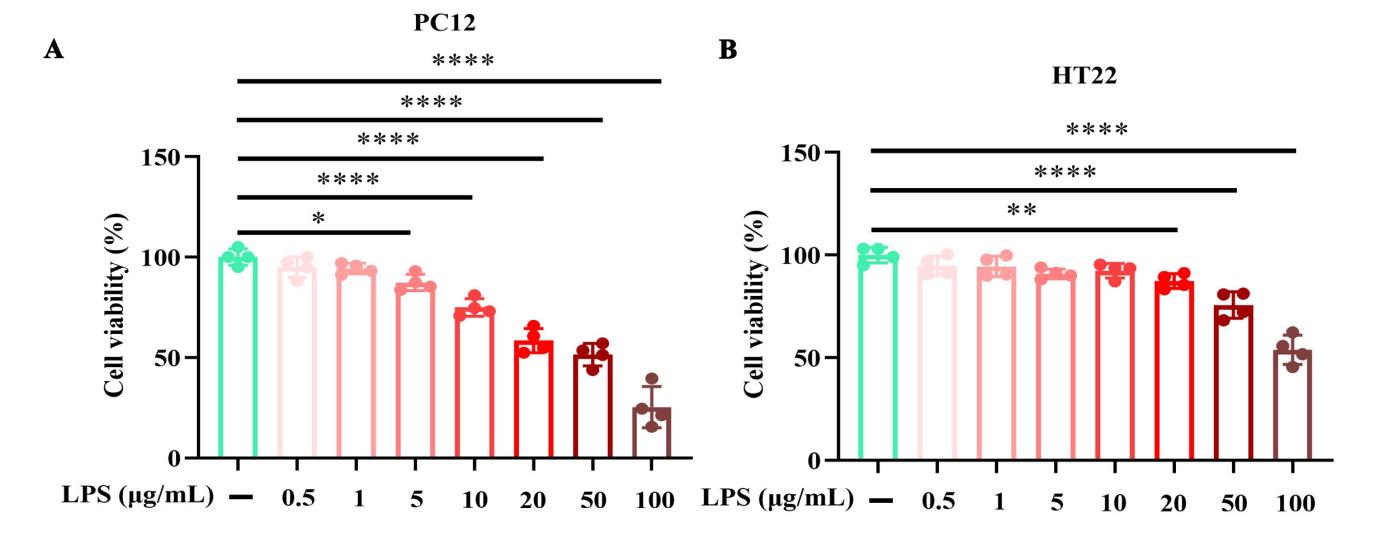
**Figure S6.** The direct effect of LPS on neuronal viability. A) The cell viability of PC12 cells (n = 4). B) The cell viability of HT22 cells (n = 4). All data are presented as mean ± SD, ^*^*P* < 0.05, ^**^*P* < 0.01, ^****^*P* < 0.0001 using GraphPad Prism 8.0 with one-way ANOVA followed by Tukey’s post hoc test.


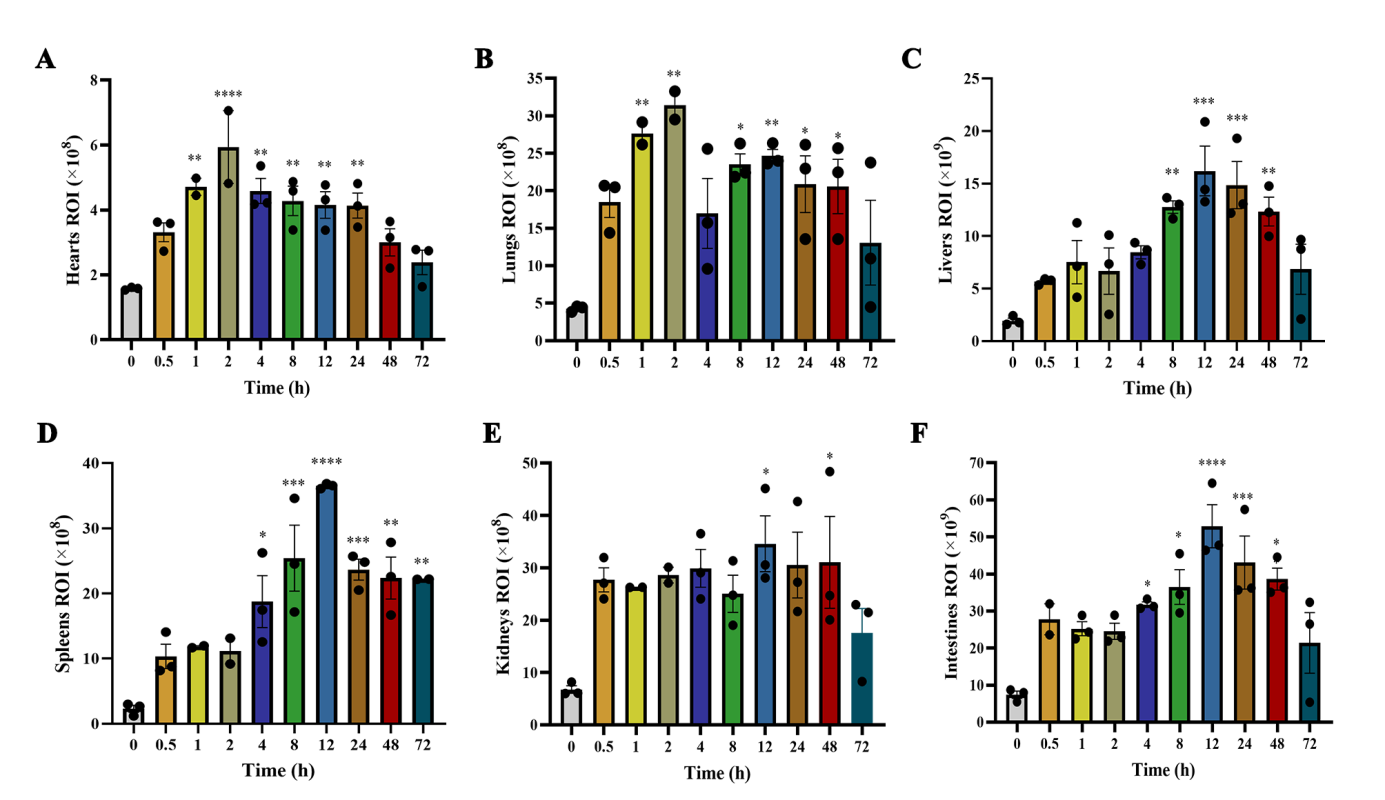


**Figrue S7.** Quantitative analysis of the accumulation of Cy5-modified Nano 18β-GA in different organs harvested from mice at different times. A) Hearts ROI (× 10^8^) (n = 3). B) Lungs ROI (× 10^8^) (n = 3). C) Lives ROI (× 10^9^) (n = 3). D) Spleens ROI (× 10^8^) (n = 3). E) Kidneys ROI (× 10^8^) (n = 3). F) Intestines ROI (× 10^9^) (n = 3). All data are presented as mean ± SEM, ^*^*P* < 0.05, ^**^*P* < 0.01, ^***^*P* < 0.001, ^****^*P* < 0.0001 using one-way ANOVA followed by Tukey’s post hoc test.


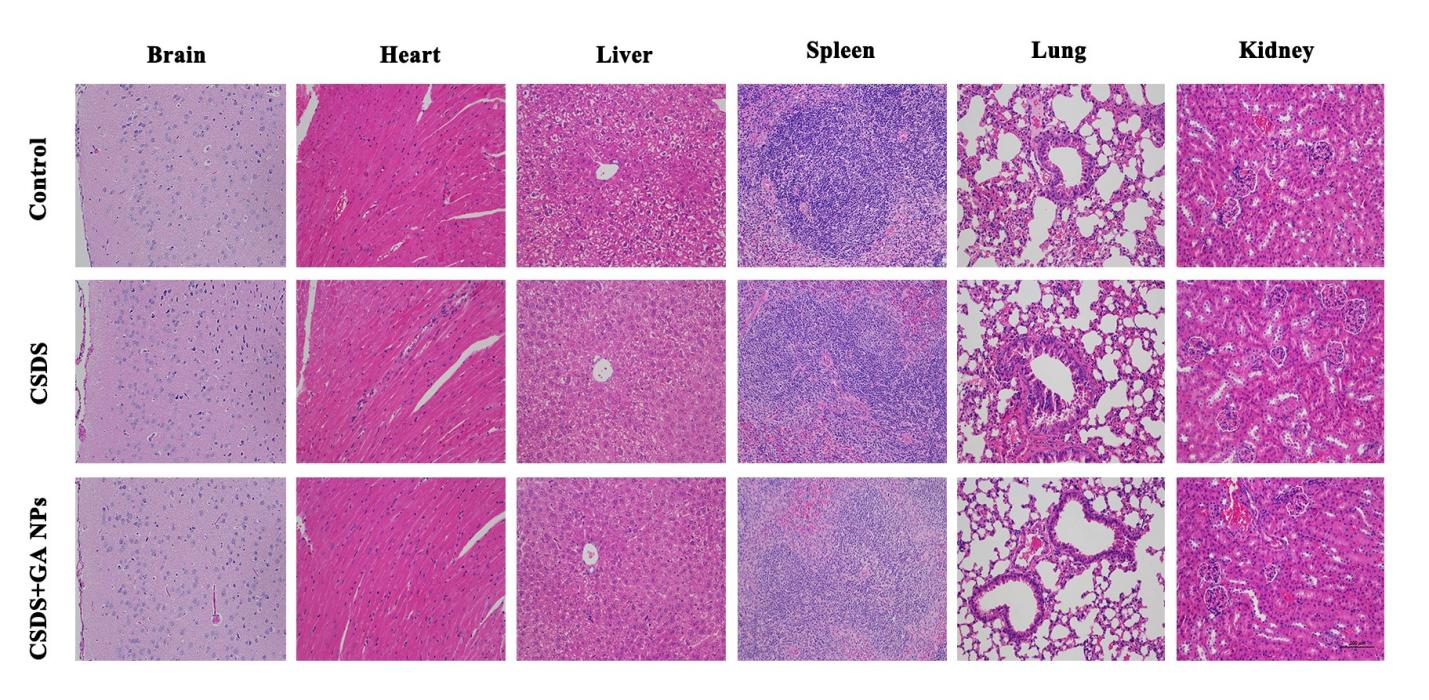


**Figure S8.** HE staining of mouse brain, heart, liver, spleen, lung, kidney (n=10). Scare bar: 100 μm.


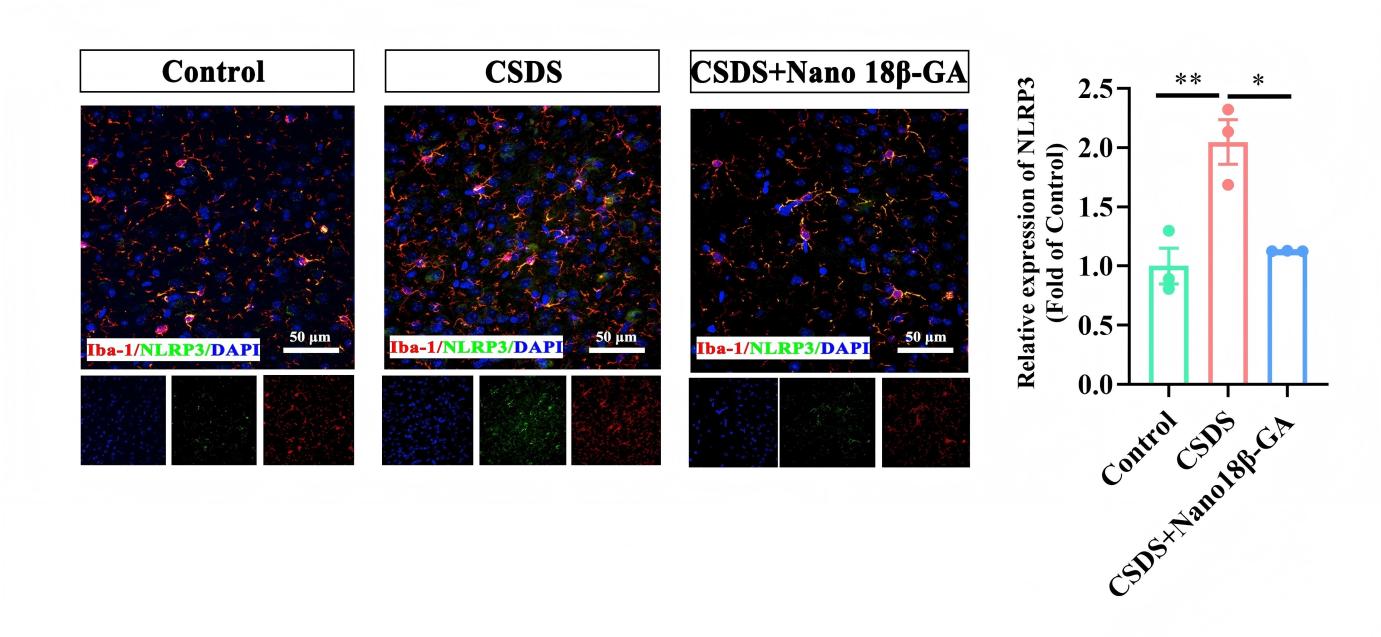


**Figure S9.** Representative immunofluorescence images and quantification of NLRP3 in the mPFC (n = 3 of randomly selected regions from 10 mice per group). Scale bar: 50 μm. Data are presented as mean ± SEM, ^*^*P* < 0.05, ^**^*P* < 0.01 using GraphPad Prism 8.0 with one-way ANOVA followed by Tukey’s post hoc test.


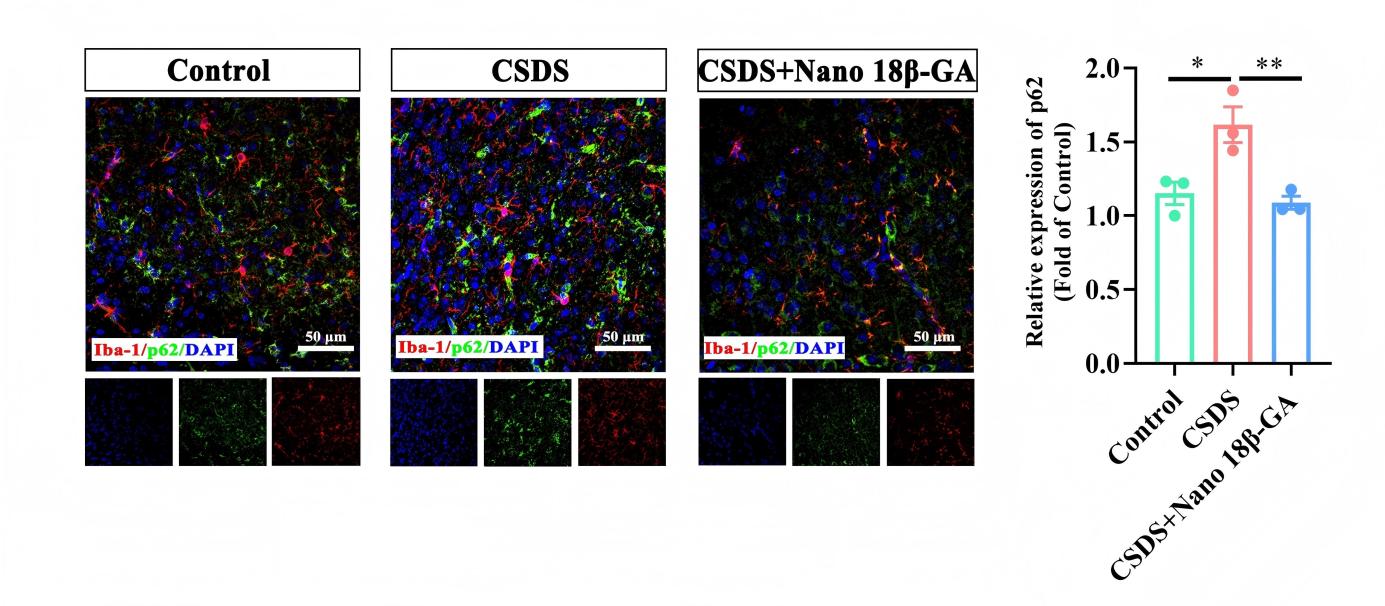


**Figure S10.** Representative immunofluorescence images and quantification of p62 in the mPFC (n = 3). Scale bar: 50 μm. Data are presented as mean ± SD, ^*^*P* < 0.05, ^**^*P* < 0.01 using one-way ANOVA followed by Tukey’s post hoc test.


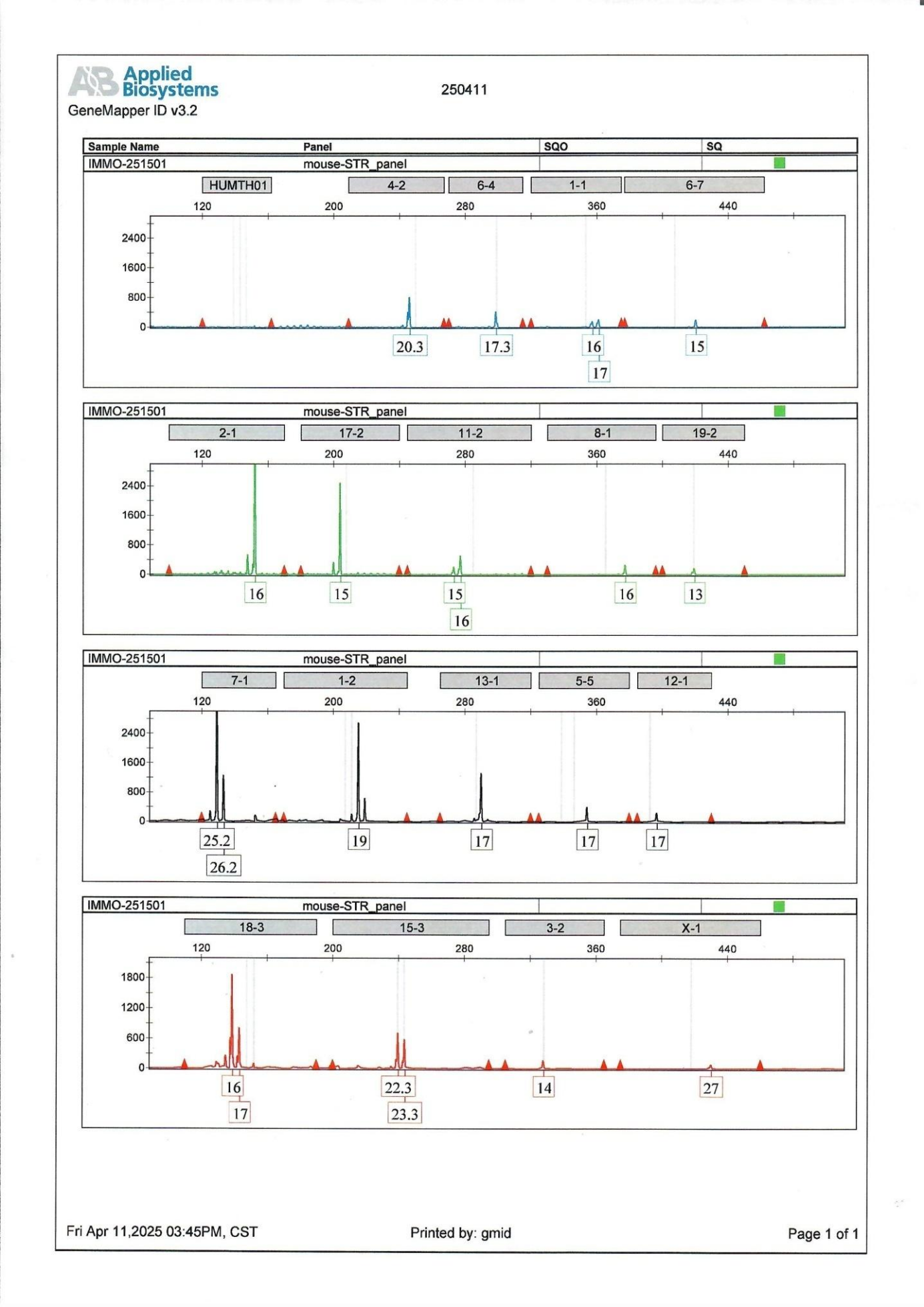


**Figure S11.** Genotyping results of the STR and Amelogenin loci of BV2 cells.


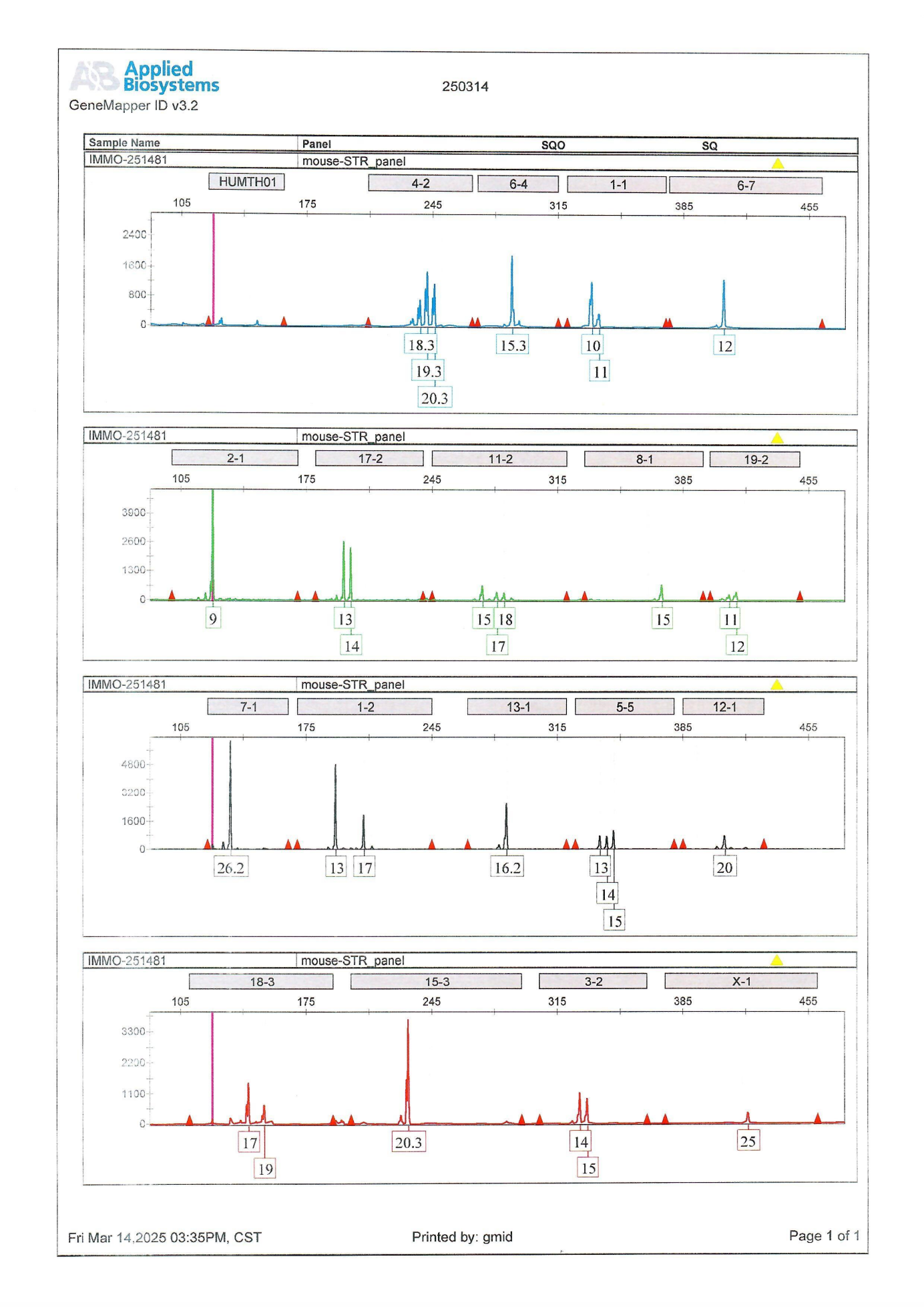
**Figure S12.** Genotyping results of the STR and Amelogenin loci of HT22 cells.


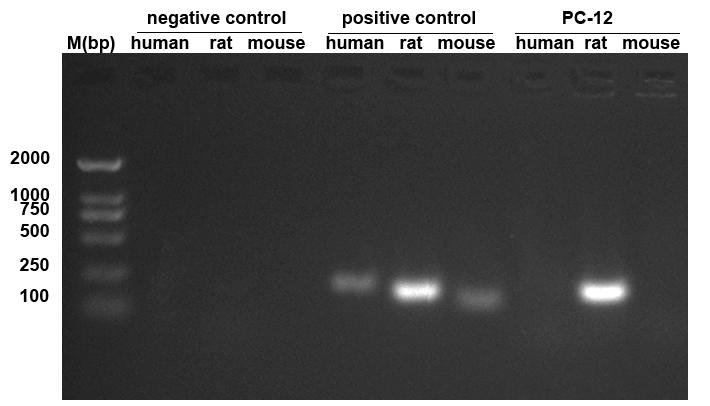


**Figure S13.** The species detected in the PC12 cell sample is rat.

**Table S1.** The binding energy by MMPBSA (kcal/mol)

| Type | mTOR-18β-GA |
| --- | --- |
| E_VDW_ | -42.41+/-1.13 |
| E_ELE_ | -16.24+/-0.58 |
| E_GB_ | 36.37+/-0.26 |
| E_SA_ | -3.26+/-0.02 |
| G_binding energy_ | -25.54+/-1.16 |

*E_VDW_*: van der Waals energy

*E_ELE_*: eletrostatic energy

*E_GB_*: polar contribution to solvation

*E_SA_*: non-polar contribution to solvation

| Loci | Allele1 | Allele2 | Allele3 | Allele1 | Allele2 | Allele3 |
| --- | --- | --- | --- | --- | --- | --- |
| TH01(Human) | - |  |  |  |  |  |
| 4-2 | 20.3 |  |  | 20.3 |  |  |
| 6-4 | 17.3 |  |  | 17.3 |  |  |
| 1-1 | 16 | 17 |  |  |  |  |
| 6-7 | 15 |  |  | 15 |  |  |
| 2-1 | 16 |  |  |  |  |  |
| 17-2 | 15 |  |  |  |  |  |
| 11-2 | 15 | 16 |  |  |  |  |
| 8-1 | 16 |  |  |  |  |  |
| 19-2 | 13 |  |  |  |  |  |
| 7-1 | 25.2 | 26.2 |  |  |  |  |
| 1-2 | 19 |  |  |  |  |  |
| 13-1 | 17 |  |  |  |  |  |
| 5-5 | 17 |  |  | 17 |  |  |
| 12-1 | 17 |  |  | 16 |  |  |
| 18-3 | 16 | 17 |  | 16 | 17 |  |
| 15-3 | 22.3 | 23.3 |  | 22.3 | 23.3 | 24.3 |
| 3-2 | 14 |  |  |  |  |  |
| X-1 | 27 |  |  | 27 |  |  |

**Table S2.** Genotyping results of the STR and Amelogenin loci of BV2 cells.

**Table S3.** Genotyping results of the STR and Amelogenin loci of HT22 cells.

| Loci | Allele1 | Allele2 | Allele3 | Allele1 | Allele2 | Allele3 |
| --- | --- | --- | --- | --- | --- | --- |
| TH01(Human) | - |  |  | - |  |  |
| 4-2 | 18.3 | 19.3 | 20.3 | 18.3 | 19.3 | 20.3 |
| 6-4 | 15.3 |  |  | 15.3 |  |  |
| 1-1 | 10 | 11 |  | 10 | 11 |  |
| 6-7 | 12 |  |  | 12 |  |  |
| 2-1 | 9 |  |  | 9 |  |  |
| 17-2 | 13 | 14 |  | 12 | 13 | 14 |
| 11-2 | 15 | 17 | 18 | 15 | 17 | 18 |
| 8-1 | 15 |  |  | 15 |  |  |
| 19-2 | 11 | 12 |  | 11 | 12 |  |
| 7-1 | 26.2 |  |  | 29 |  |  |
| 1-2 | 13 | 17 |  | 13 | 17 |  |
| 13-1 | 16.2 |  |  | 16.2 |  |  |
| 5-5 | 13 | 14 | 15 | 13 | 14 | 15 |
| 12-1 | 20 |  |  | 20 |  |  |
| 18-3 | 17 | 19 |  | 17 | 19 |  |
| 15-3 | 20.3 |  |  | 20.3 |  |  |
| 3-2 | 14 | 15 |  | 14 | 15 |  |
| X-1 | 25 |  |  | 25 |  |  |

**Table S4.** Detailed information on all antibodies used in this study.

| Antigen | Source | Clonality | Cat | Manufacturer (city, state, country) |
| --- | --- | --- | --- | --- |
| Anti-Iba1 antibody [EPR16589] – Mouse IgG1 (Chimeric) | Mouse | monoclonal | ab283319 | Abcam (Cambridge,Cambs, UK) |
| Anti-NLRP3 Antibody | Rabbit | Polyclonal | BA3677 | [BOSTER (](http://boster.com/)Wuhan, Hubei, CN) |
| Anti-iNOS Rabbit pAb | Rabbit | Polyclonal | GB11119 | [Servicebio (](http://boster.com/)Wuhan, Hubei, CN) |
| Anti-SQSTM1/p62 Rabbit pAb | Rabbit | Polyclonal | GB11531 | [Servicebio (](http://boster.com/)Wuhan, Hubei, CN) |
| Anti-Caspase-1 antibody [EPR19672] | Rabbit | monoclonal | ab207802 | Abcam (Cambridge,Cambs, UK) |
| Arginase-1 (D4E3M™) XP® Rabbit mAb | Rabbit | Monoclonal | 93668T | Cell Signaling Technology (Beverly, MA, USA) |
| Goat Anti-Mouse IgG H&L (Alexa Fluor® 488) | Goat | Polyclonal | ab150113 | Abcam (Cambridge,Cambs, UK) |
| Goat Anti-Rabbit IgG H&L (Alexa Fluor® 594) | Goat | Polyclonal | ab150080 | Abcam (Cambridge,Cambs, UK) |
| LC3B Antibody | Rabbit | Polyclonal | 2775 | Cell Signaling Technology (Beverly, MA, USA) |
| mTOR Antibody | Rabbit | Polyclonal | 2972T | Cell Signaling Technology (Beverly, MA, USA) |
| Phospho-mTOR (Ser2448) (D9C2) XP® Rabbit mAb | Rabbit | Monoclonal | 5536T | Cell Signaling Technology (Beverly, MA, USA) |
| p70 S6 Kinase (E8K6T) XP® Rabbit mAb | Rabbit | Monoclonal | 34475T | Cell Signaling Technology (Beverly, MA, USA) |
| Phospho-p70 S6 Kinase (Thr389) (108D2) Rabbit mAb | Rabbit | Monoclonal | 9234T | Cell Signaling Technology (Beverly, MA, USA) |
| Rabbit anti-NLRP3 Polyclonal Antibody | Rabbit | Polyclonal | abs151715 | Absin (Shanghai,Shanghai,CN) |
| Rabbit anti-ASC Polyclonal Antibody | Rabbit | Polyclonal | abs155599 | Absin (Shanghai,Shanghai,CN) |
| SQSTM1/p62 (D6M5X) Rabbit mAb | Rabbit | Monoclonal | 23214T | Cell Signaling Technology (Beverly, MA, USA) |
| β-Actin (8H10D10) Mouse mAb | Mouse | Monoclonal | 3700T | Cell Signaling Technology (Beverly, MA, USA) |
| Anti-mouse IgG, HRP-linked Antibody | Horse | / | 7076T | Cell Signaling Technology (Beverly, MA, USA) |
| Anti-rabbit IgG, HRP-linked Antibody | Goat | / | 7074T | Cell Signaling Technology (Beverly, MA, USA) |
